# Between the Vinča and *Linearbandkeramik* Worlds: The Diversity of Practices and Identities in the 54th–53rd Centuries cal BC in Southwest Hungary and Beyond

**DOI:** 10.1007/s10963-016-9096-x

**Published:** 2016-09-08

**Authors:** János Jakucs, Eszter Bánffy, Krisztián Oross, Vanda Voicsek, Christopher Bronk Ramsey, Elaine Dunbar, Bernd Kromer, Alex Bayliss, Daniela Hofmann, Peter Marshall, Alasdair Whittle

**Affiliations:** 1 Institute of Archaeology, Research Centre for the Humanities, Hungarian Academy of Sciences, Úri utca 49, 1014 Budapest, Hungary; 2 Römisch-Germanische Kommission, Palmengartenstraße 10–12, 60325 Frankfurt a. M., Germany; 3 Barátúr utca 9, 7625 Pécs, Hungary; 4 Oxford Radiocarbon Accelerator Unit, Research Laboratory for Archaeology and the History of Art, University of Oxford, Dyson Perrins Building, Oxford, OX1 3QY UK; 5 SUERC Radiocarbon Dating Laboratory, Scottish Enterprise Technology Park, Rankine Avenue, East Kilbride, G75 0QF UK; 6 Klaus-Tschira-Labor, Curt-Engelhorn-Zentrum Archaeometrie, C 5 Zeughaus, 68159 Mannheim, Germany; 7 Historic England, 1 Waterhouse Square, 138–142 Holborn, London, EC1N 2ST UK; 8 Institute of Archaeology, University of Hamburg, Edmund-Siemers-Allee 1, Flügel West, 20146 Hamburg, Germany; 9 Department of Archaeology and Conservation, Cardiff University, John Percival Building, Colum Drive, Cardiff, CF10 3EU UK

**Keywords:** Neolithic, Transdanubia, Formal chronological modelling, Longhouses, Material diversity, Identities

## Abstract

Perhaps nowhere in European prehistory does the idea of clearly-defined cultural boundaries remain more current than in the initial Neolithic, where the southeast–northwest trend of the spread of farming crosses what is perceived as a sharp divide between the Balkans and central Europe. This corresponds to a distinction between the Vinča culture package, named for a classic site in Serbia, with its characteristic pottery assemblage and absence of longhouses, and the *Linearbandkeramik* (LBK), with equally diagnostic but different pottery, and its apparently culturally-diagnostic longhouses, extending in a more northerly belt through central Europe westward to the Dutch coast. In this paper we question the concept of such a clear division through a presentation of new data from the site of Szederkény-Kukorica-dűlő. A large settlement in southeast Transdanubia, Hungary, excavated in advance of road construction, Szederkény is notable for its combination of pottery styles, variously including Vinča A, Ražište and LBK, and longhouses of a kind otherwise familiar from the LBK world. Formal modelling of its date establishes that the site probably began in the later 54th century cal BC, lasting until the first decades of the 52nd century cal BC. Occupation, featuring longhouses, pits and graves, probably began at the same time in the eastern and western parts of the settlement, starting a decade or two later in the central part; the western part was probably the last to be abandoned. Vinča pottery is predominantly associated with the eastern and central parts of the site, and Ražište pottery with the west. Formal modelling of the early history of longhouses in the LBK world suggests their emergence in the Formative LBK of Transdanubia c. 5500 cal BC followed by rapid dispersal in the middle of the 54th century cal BC, associated with the ‘earliest’ (*älteste*) LBK. The adoption of longhouses at Szederkény thus appears to come a few generations after the start of this ‘diaspora’. Rather than explaining the mixture of things, practices and perhaps people at Szederkény with reference to problematic notions such as hybridity, we propose instead a more fluid and varied vocabulary, encompassing combination and amalgamation, relationships and performance in the flow of social life, and networks; this makes greater allowance for diversity and interleaving in a context of rapid change.

## Absztrakt

Talán nincs még egy olyan időszak és terület az európai neolitikum kutatásában, ahol a kultúrák közötti éles határok megkülönböztetése annyira hangsúlyos lenne, mint a földművelés terjedésének az a határvidéke, amelyet a Balkán és Közép-Európa közötti éles választóvonalként értelmeznek. Ez megfelel annak a határozott különbségtételnek, amellyel a sajátos kerámiaművessége alapján elkülönített, szerbiai névadó lelőhelye alapján Vinča-ként ismert kulturális egységet, és az tőle északra, Közép-Európán át nyugat felé egészen a holland partokig elterjedt és ugyancsak sajátos kerámiastílussal és egyedinek tűnő hosszúházakkal jellemezhető vonaldíszes kerámia kultúrát (közép-európai VK) választják el egymástól.Tanulmányunk a délkelet-dunántúli (Magyarország) Szederkény-Kukorica-dűlő lelőhely vizsgálatának új eredményei alapján ezt a fajta éles különbségtételt veszi górcső alá. Az autópálya építését megelőző feltárás során napvilágra került nagyméretű neolitikus település egyrészt a vonaldíszes kultúrára jellemző hosszúházai, másrészt sajátos összetételű, korai Vinča, Ražište és vonaldíszes stílusú kerámiát is tartalmazó kerámia leletegyüttesei miatt figyelemre méltó. Az adatok formális modellje alapján megállapítható, hogy a lelőhely élete a Kr.e. 54. évszázad fiatalabb szakaszában kezdődhetett, és valószínűleg a Kr.e. 52. évszázad első néhány évtizedéig tartott. A megtelepedés, amelyet épületek, gödrök és temetkezések jeleznek, egyidejűleg kezdődhetett a település keleti és nyugati részén, míg a középső településrész egy vagy két évtizeddel később létesült. A vizsgálat azt is megállapította, hogy minden bizonnyal a nyugati településrészt hagyták el legkésőbb. A korai Vinča stílusú kerámia túlnyomórészt a keleti és középső településrészekkel hozható összefüggésbe, míg a Ražište stílus inkább a nyugati településrészen meghatározó. A hosszúházak korai történetére vonatkozó formális modell alapján az épülettípus megjelenése a dunántúli formatív vonaldíszes fázisban, Kr.e. 5500 körül feltételezhető, majd nem sokkal azután, a Kr.e. 54. évszázad derekán (a kultúra Kárpát-medencétől nyugatra legkorábbinak nevezett, ’älteste’ szakaszában) gyors terjedésnek indult. Szederkényben az első hosszúházak ennek megfelelően, néhány generációval a közép-európai VK diaszpóráját követően épülhettek. Ahelyett, hogy a különféle tárgyi leletek, szokások és esetlegesen embercsoportok keveredésének magyarázatára olyan problematikus fogalmakat használnánk, mint amilyen a ’hibriditás’, ennél sokoldalúbb és változatosabb fogalomrendszert vezetünk be, amely a szociális hálózatokban zajló olyan folyamatok, mint az összeolvadás, keveredés és kapcsolatok különféle mechanizmusait is magában foglalja. Ennélfogva nagyobb teret enged a sokféleségnek és az átfedéseknek egy gyorsan változó közegben.

## Introduction: Separate Worlds or Interleaved Networks?

**Fig. 1:**
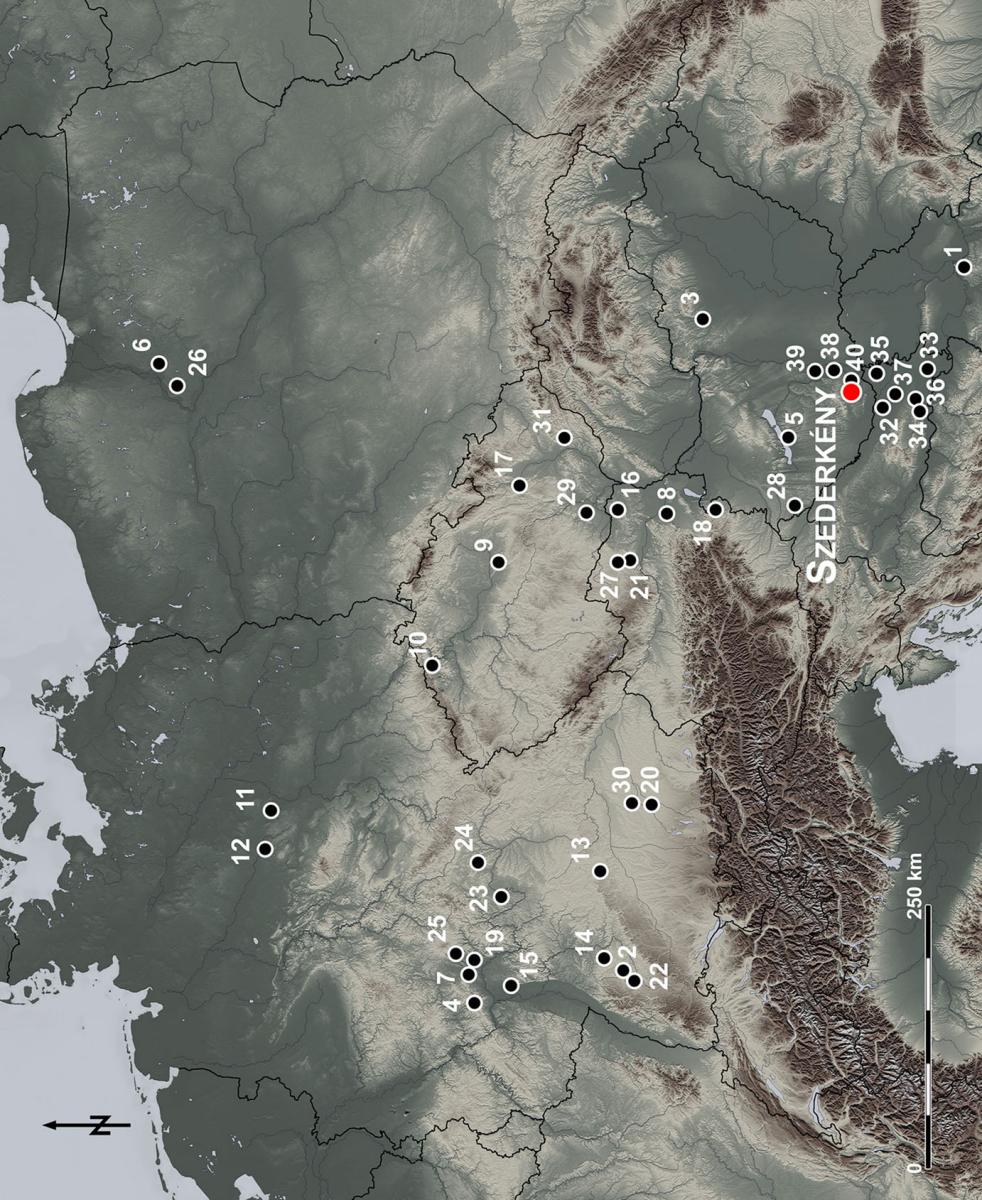
Map showing the location of sites discussed in the text (including those with radiocarbon dates that have been incorporated in the chronological models presented). Site with Vinča pottery style: *1*—Vinča-Belo Brdo. Sites with formative and earliest LBK pottery style: *2*—Ammerbach-Pfäffingen Lüsse; *3*—Apc-Berekalja I; *4*—Bad Camberg-Würges; *5*—Balatonszárszó-Kis-erdei-dűlő; *6*—Boguszewo 41; *7*—Bruchenbrücken; *8*—Brunn/Wolfholz; *9*—Bylany; *10*—Chabařovice; *11*—Eilsleben; *12*—Eitzum; *13*—Enkingen; *14*—Gerlingen; *15*—Goddelau; *16*—Kleinhadersdorf; *17*—Mohelnice; *18*—Neckenmarkt; *19*—Nidderau-Ostheim; *20*—Niederhummel; *21*—Rosenburg; *22*—Rottenburg-Fröbelweg; *23*—Schwanfeld; *24*—Stadel; *25*—Steinfurth Bad Nauheim; *26*—Stolno; *27*—Strögen; *28*—Szentgyörgyvölgy-Pityerdomb; *29*—Vedrovice; *30*—Wang; *31*—Žopy. Sites with early Sopot/Ražište pottery style: *32*—Donji Miholjac, Golinci; *33*—Dubovo-Košno; *34*—Ivandvor; *35*—Kneževi Vinogradi-Osnovna škola; *36*—Krčavina-Novi Perkovci; *37*—Podgorač-Ražište. Sites with early Vinča (A1–A3) and LBK pottery styles: *38*—Alsónyék-Bátaszék; *39*—Tolna-Mözs. Site with early Vinča (A1–A3), early LBK and early Sopot/Ražište pottery styles: *40*—Versend-Gilencsa

A century or more of research has established the outlines of the major Neolithic developments in the Carpathian basin and central Europe. By the second half of the sixth millennium cal BC, in culture-historical terms, there were two major groupings across this broad area: the Vinča culture to the south and the *Linearbandkeramik* (LBK) to the north (Fig. 1). The Vinča culture represents further development, following beginnings in the late seventh and early sixth millennia cal BC, while the LBK stands for the first Neolithic activity in central Europe; early Neolithic Starčevo predecessors in western Hungary or Transdanubia, Croatia and Serbia are to be noted. In general terms, these two major phenomena have tended to be kept apart, and there are certainly separate research communities investigating them. The Vinča world had tells among its settlement repertoire, and distinctive material culture including black- and red-fired pottery, anthropomorphic lids and figurines, while the LBK world is well known for its post-framed timber longhouses and band-decorated, fine ware pottery. Only two sites with burials are certainly known in the Vinča orbit (and only one of these, Botoš, is of early Vinča date), while many settlement burials and burial grounds are known from the LBK, especially from its more developed phases. Finally, different origins have been proposed, many authors in the past having looked far south for Vinča origins, while more recent research has looked to the late Starčevo culture in Transdanubia as a likely candidate for LBK beginnings (Chapman 1981; Bánffy 2004; Brukner and Vorgić 2006; Borić 2009; Bánffy and Oross 2010; Bickle and Whittle 2013).

The boundary between these two networks would conventionally be drawn somewhere in the regions of northernmost Croatia and Serbia, in the northern Banat and in Vojvodina, and in southernmost Hungary, both in southeast Transdanubia and the southern Alföld (e.g. Chapman 1981, fig. 13; Markotić 1984, map 2; Tringham and Krstić 1990, fig. 16.1; Horváth 2006; Paluch 2011) (Fig. 1). Our description so far, however, reflects the use of the culture concept, which, while useful in pragmatic terms for ordering and making sense of diverse evidence, tends to reinforce long-held notions of fixed and bounded identities. The concepts of stable identities and sharp boundaries should be challenged and questioned. From a theoretical point of view, the danger of rigid categorisations has recently been stated by Andrew Jones: ‘One of the consequences of categorization is that artefacts are conceptualized as static things or objects; they are circumscribed by their categories and the material components of categories are equally held in stasis or circumscribed’ (Jones 2012, pp. 189–190).

Interesting choices follow from this kind of perspective. If the notion of separate cultures is retained, variations have to be covered by notions of mixture, including hybridity, many of which are problematic because they again rest on concepts of separation and boundedness (Borić 2005; Ingold and Hallam 2007). In a nutshell, as Philipp Stockhammer (2012, p. 2) has put it, ‘every discipline which argues about hybridity has to define what it understands to be pure’ (cf. Liebmann 2015; Silliman 2015; Voss 2015). If, however, a more fluid and varied vocabulary is adopted, including combination and amalgamation; relationships and performance in the flow of social life; and interaction spheres, networks and even meshworks (Caldwell 1955; Latour 1993; Ingold 2011), much greater allowance can be made for diversity and interleaving.

Hungarian prehistorians have in fact already drawn attention to an area within southeast Transdanubia where things and practices have been found in what are from a conventional, culture-historical point of view unusual combinations. Discoveries from the early to the late Neolithic periods (from the early sixth to the first half of the fifth millennium cal BC) in southeastern Transdanubia have long shown the particularly important role of the region, along the right (west) bank of the Danube, as an intermediate zone between the Balkans and central Europe. Recent research at the site of Szederkény-Kukorica-dűlő in this part of southwest Hungary brings these themes into particularly sharp focus, due to the joint presence of a ceramic repertoire which includes Vinča pottery (and a variant called the Ražište style) and longhouse architecture characteristic of the LBK world. Key features of the development of the Neolithic in southeast Transdanubia, of the relevant pottery styles across a broader area, and of the emergence of longhouse architecture, must first briefly be introduced.

## Cultural Sequences: An Outline

### The LBK Sequence

We now know that the first farming communities in Transdanubia, labelled the Starčevo culture and thought to have come from the northern Balkans (Kalicz 1990), went as far north as the region of Lake Balaton (Simon 1996; Bánffy 2006; Regenye 2007, 2010). Alsónyék-Bátaszék in southeast Transdanubia stands out as altogether exceptional, with more than 500 features, though without definite evidence for the nature of houses (Bánffy et al. 2010), and it matches the scale of Starčevo sites in the core area of Slavonia (northern Croatia) and Serbia. Further finds in motorway and other projects help to suggest a rather dense Starčevo settlement network in the first half of the sixth millennium cal BC.

Clear evidence of a Starčevo–LBK transition within Transdanubia was established at Szentgyörgyvölgy-Pityerdomb, out to the west in the Kerka valley close to the modern border with Slovenia (Bánffy 2004, 2013b). Here two longhouses were found, with an arguably general resemblance to LBK-type buildings. There was also a flint assemblage with close comparisons to late Mesolithic lithic technology and typology (cf. T. Biró 2005; Mateiciucová 2008), but the Pityerdomb pottery—apart from 0.5% (some hundred sherds) with incised linear decorations—can be considered almost entirely as of late Starčevo character. Other sites in central Transdanubia in the region of Lake Balaton may also be added to this ‘missing link’ between Starčevo and LBK, now proposed as the Formative LBK phase (Bánffy 2000, 2004; Bánffy and Oross 2009, 2010). An early LBK phase follows, with Bicske-Bíňa and Milanovce phase subdivisions, tentatively proposed as starting at c. 5450 cal BC based on results from eastern Austria (Lenneis and Lüning 2001; Lenneis and Stadler 2002; Oross and Bánffy 2009, p. 182, table 1; Lenneis 2010) or a little later, around 5400 cal BC (Stadler and Kotova 2010, p. 338). Late LBK, from c. 5300/5250 cal BC, is labelled Notenkopf and Zseliz/Želiezovce in northern Transdanubia and Keszthely in central and southern Transdanubia (Oross and Bánffy 2009, p. 185, table 1). By the time of the late LBK in these Transdanubian terms, substantial settlements, such as Balatonszárszó-Kis-erdei-dűlő, are known, with developed longhouses which relate firmly to the architecture of central Europe and beyond (Oross 2010, pp. 65–71, figs. 7.1–7.7; Marton and Oross 2012; Oross 2013a).

LBK pottery assemblages had also been tentatively connected with ideas of some kind of Balkan impact or influence, from the early phases of the Vinča culture (e.g. Kalicz and Makkay 1972; Kalicz 1980, 1994; Makkay 1982). In discussion of Bicske in northern Transdanubia, possible links with the Vinča world were further emphasised (Makkay 1978). These putative connections were also subsequently discussed in relation to LBK sites on the left (east) bank of the Danube, such as Fajsz-Garadomb and Bajaszentistván, as well as in summaries of the state of Transdanubian LBK research (Kalicz 1993, 1994). The possibility of some Vinča ‘penetration’ into different regions of the Danube valley was also suggested (Horváth 2006; Marton and Oross 2012; Jakucs and Voicsek 2015). Did these Vinča-style things—principally pots—represent a cultural ‘impact’, imported wares, or the established presence of new people, with settlement sites to prove it? These distinctions have rarely been spelled out, and their implications have rarely been thought through.

**Fig. 2:**
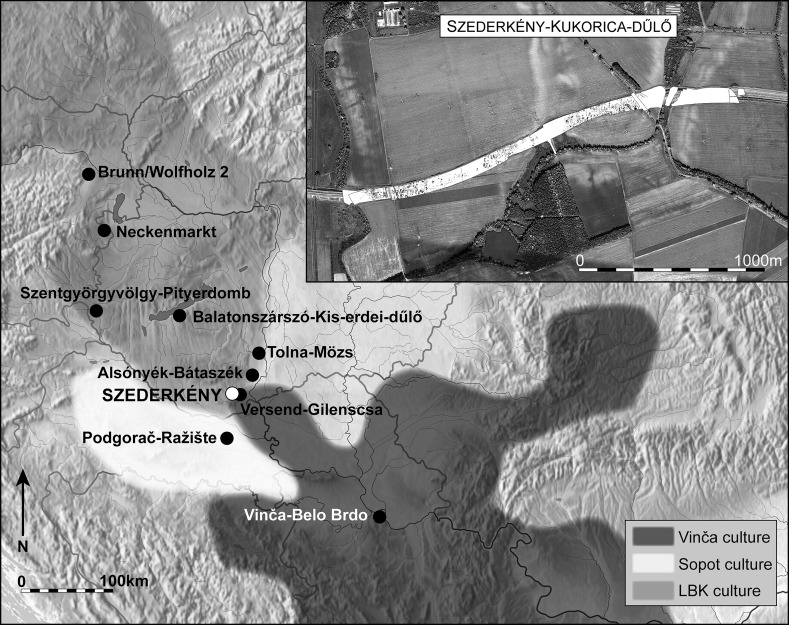
Map showing the location of Szederkény and nearby sites, and the maximum spatial distributions of the ceramic traditions present in the region in the last centuries of the sixth millennium cal BC

**Fig. 3:**
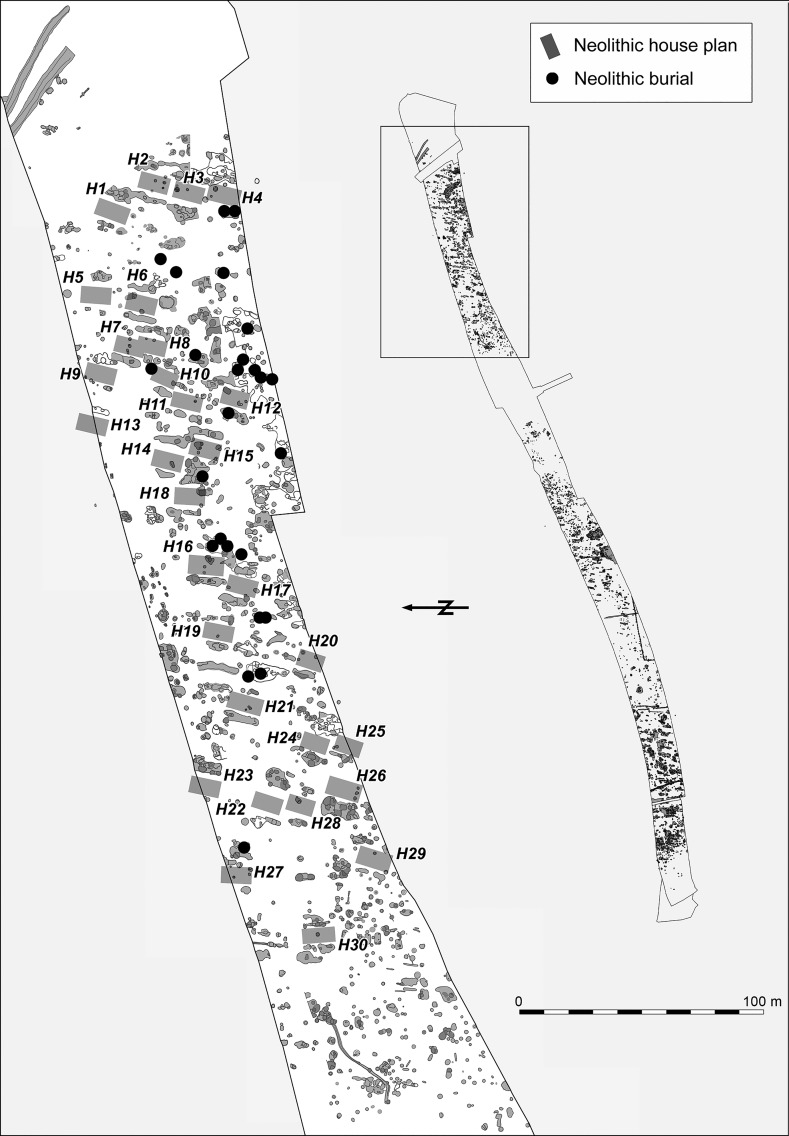
Layout of the eastern part of the settlement

**Fig. 4:**
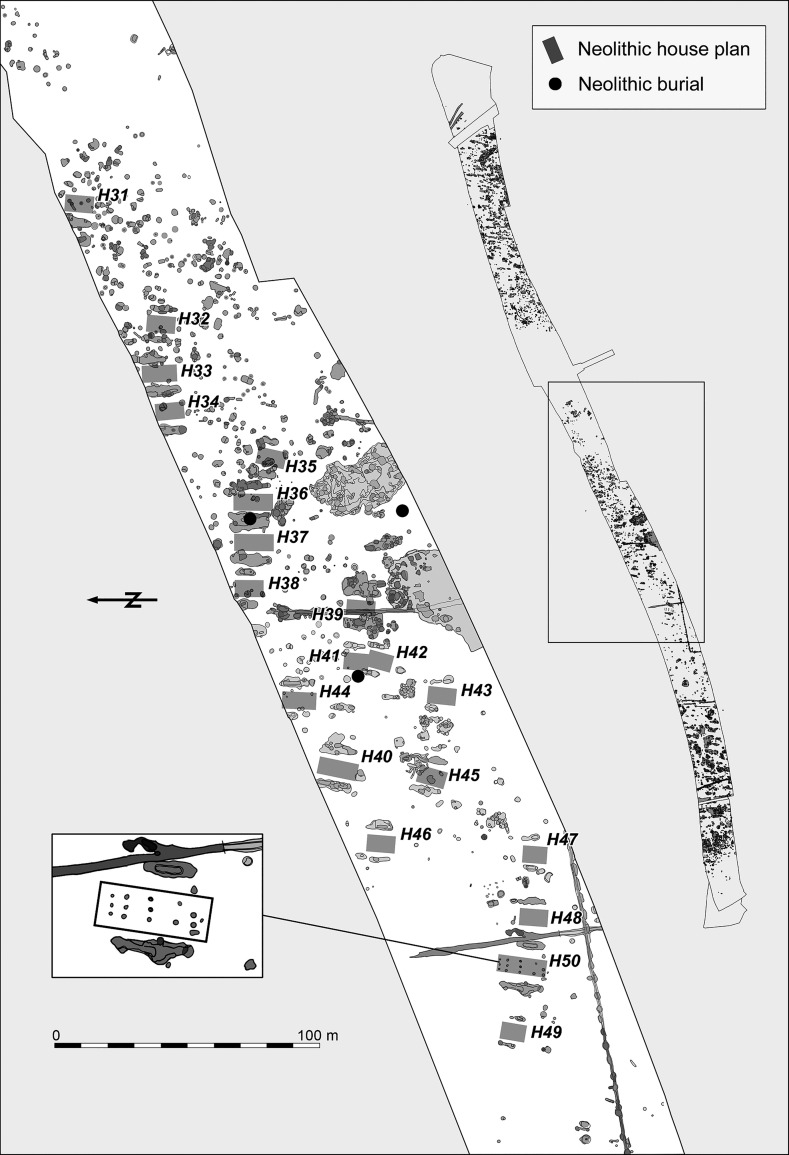
Layout of the central part of the settlement. House 50, with better than usual preservation of internal postholes, is given in the inset

**Fig. 5:**
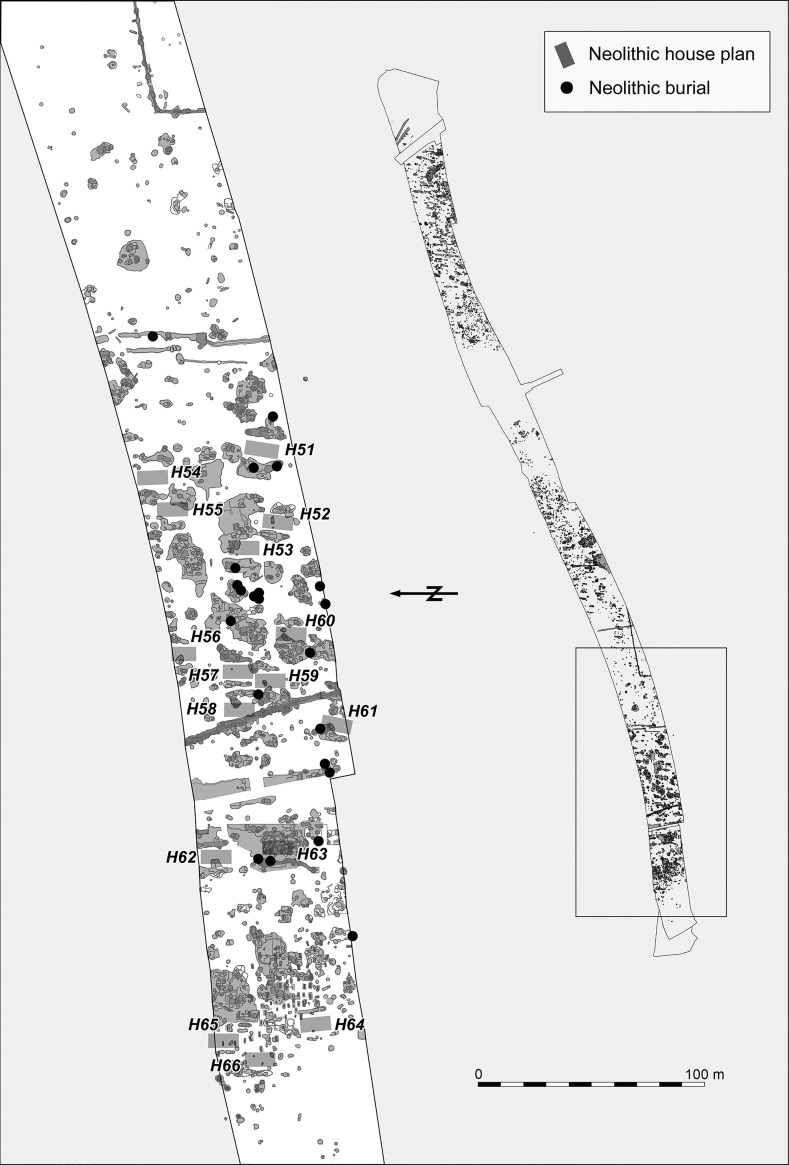
Layout of the western part of the settlement

**Fig. 6:**
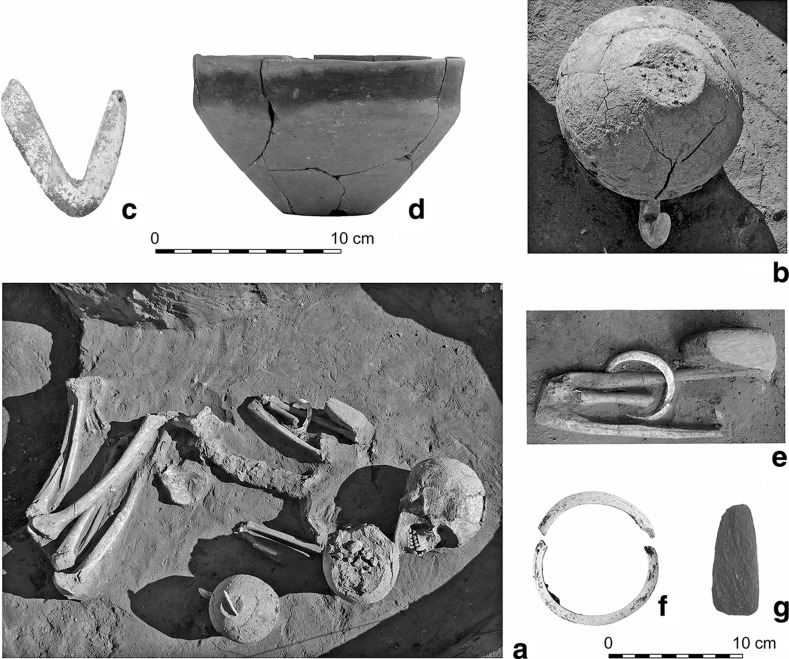
Grave 2484 (in the eastern part of the settlement). The black-topped pot is in early Vinča style

**Fig. 7:**
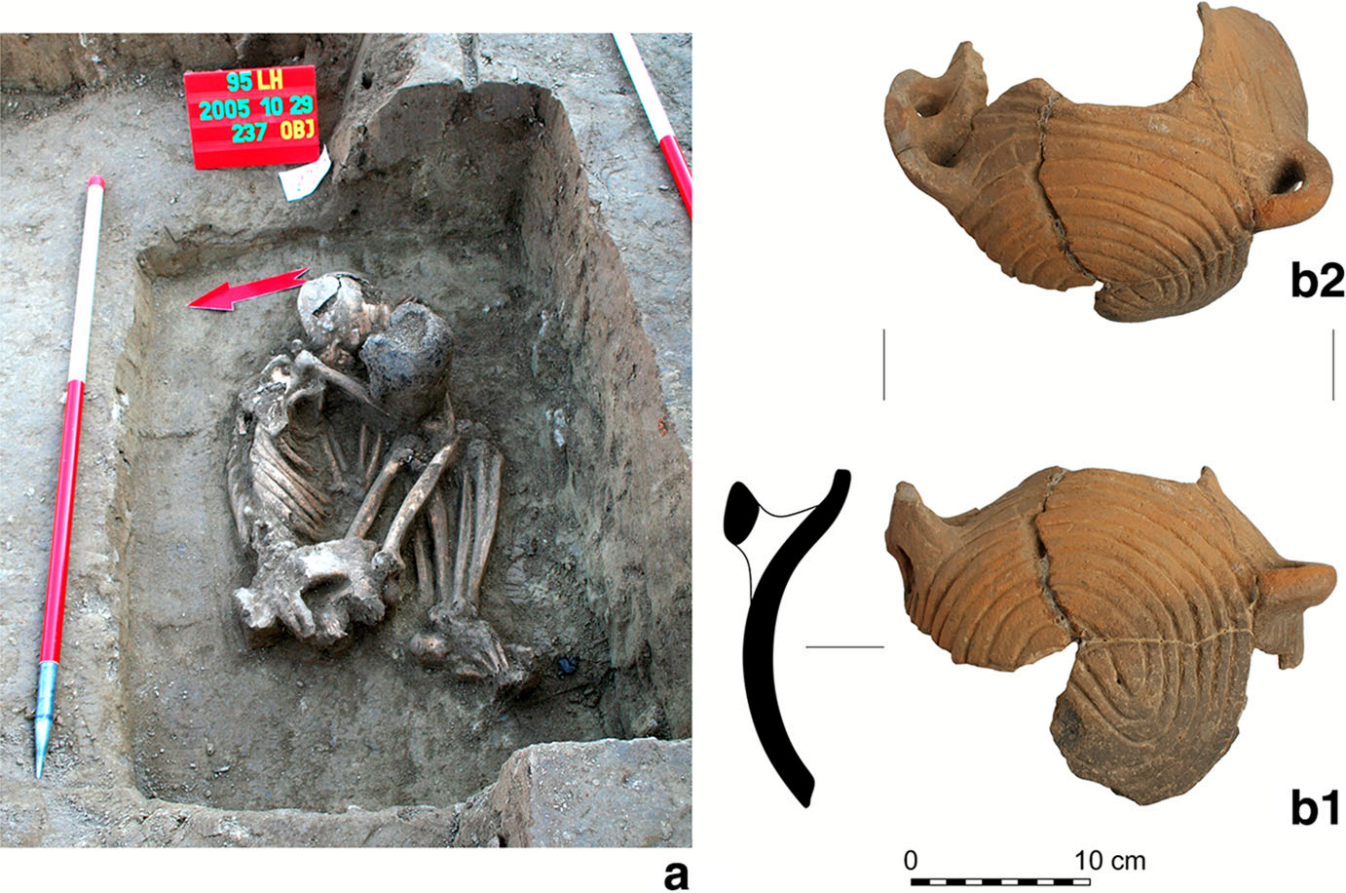
Grave 237 (in the eastern part of the settlement). The pot is in early LBK style

**Fig. 8:**
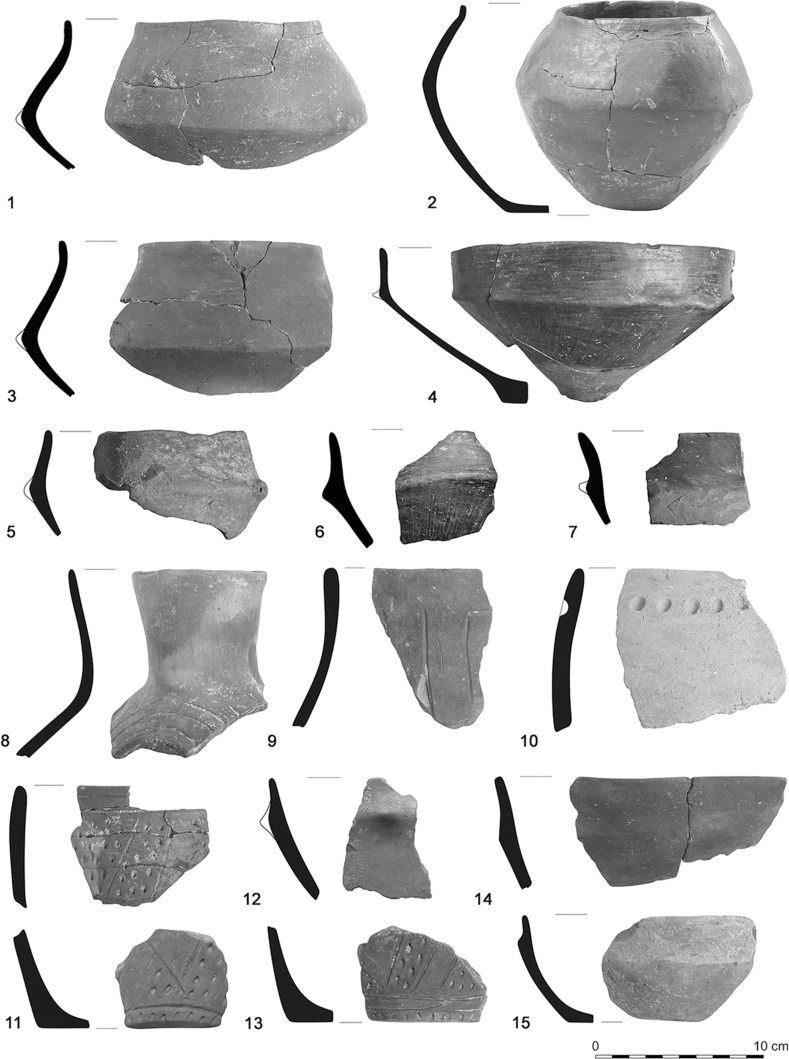
Early Vinča-style (*1*–*7*, *10*–*15*) and early LBK-style (*8*–*9*) pottery from the eastern and central parts of the settlement. *1*–*7*—House H16/Feature 316; *8*, *9*, *11*, *12*, *13*—House H36-H37/Features 1565, 1495 (different parts of the overlapping longpits between the two Houses), *10*, *13*—House H37/Features 1690, 1701; *15*—House H36/Feature 1551; *14*—House H34/Feature 1341

A site with varying proportions of early Vinča- and LBK-style material in some features, and with a layout and buildings showing LBK characteristics, was then found only a few years ago. This is the settlement of Tolna-Mözs, on the western side of the Danube near Szekszárd in the Tolna Sárköz area, and about 50 km north of Szederkény. This has three excavated parts with groups of longhouses of a kind well known from the LBK world (Marton and Oross 2012, fig. 3). In the southern part, a considerable amount of the pottery shows early Vinča characteristics, although its fabrics are not identical with those of Szederkény or assemblages south of Transdanubia. There were also sherds with strong resemblances to the latest Starčevo and earlier LBK traditions, while the assemblages of the central and the northern areas contained material both of LBK (Bicske-Bíňa and Notenkopf) and Vinča style (Marton and Oross 2012, pp. 227–232, figs. 5–8). Recent geomagnetic survey provided further evidence, however, that the settlement is more extensive, and its structure more complex, than previously expected. Numerous additional settlement nuclei have also been discovered (Rassmann et al. 2015, pp. 1–4, figs. 2–5).

### The Vinča Sequence

Overall, the broad distribution of the Vinča culture extends through the river valleys—the Danube, its tributaries and their catchments—of the northern and central Balkans, from easternmost Croatia through Serbia down to Kosovo and parts of Macedonia and Bulgaria, and from Croatia and Bosnia-Herzegovina eastwards as far as parts of Transylvania in Romania. The presence of early Vinča pottery at sites like Szederkény extends the distribution into southernmost Hungary; Vinča characteristics are an important component further to the north at sites such as Tolna-Mözs; and there is even sporadic evidence for early Vinča-style pots as far north as Bicske near Budapest (Makkay 1978). The predecessor of the Vinča culture across the northern part of its distribution was the Starčevo culture, though, as mentioned above, many past researchers have sought to derive it from much further south. The Vinča culture or network broadly belongs to the latter part of the sixth millennium cal BC and the first half of the fifth millennium cal BC (Borić 2009; Orton 2012; Porčić 2011; Tripković 2011). The formally modelled sequence at the tell site of Vinča-Belo Brdo begins in the generation after 5300 cal BC (Tasić et al. in press; cf. Schier 1996; Borić 2009, 2015). In classic terminology, early Vinča pottery has been labelled Vinča A at Vinča-Belo Brdo, between the depths of 9.3 and 8 m, and in its surrounds (Schier 1995, 1996; Tasić et al. in press; and references); this has been modelled for Belo Brdo as lasting from *5300*–*5270* *cal BC to 5200*–*5165* *cal BC* (*95% probability*; Tasić et al. in press, table 8). Early Vinča pottery has distinctive black- and red-fired fabrics, and a range of forms including various kinds of bowl, pedestalled vessels, bowls and dishes with protomes, amphorae of various kinds and sizes, some with accompanying lids, miniature vessels, fired clay ‘altars’ (footed dishes) and strainers; some decoration occurs (Schier 1996; Tasić et al. in press). On the northwest fringes of the Vinča orbit, in northeastern Croatia, a local cultural variant has been identified, known as the Sopot culture. Understanding both the formation of the Sopot culture and its chronology has long been problematic (Dimitriejević 1968; Marković 1994; Burić 2015; Jakucs and Voicsek 2015; Oross et al. in press a), but it might plausibly be linked to the spread of the Vinča culture and Transdanubian LBK influence (Dimitrijević 1968; Težak-Gregl 1993).

As is well known, both tells and flat settlements are found in the Vinča orbit, although very little is known about houses on early Vinča flat sites. On Vinča tells and in later Vinča flat sites, the houses are different to those of the LBK world, being rectangular or squarish, with walls variously defined by post-framing. These were shorter buildings than those of the LBK, lacked longpits flanking their long sides, had more visible internal furnishings, and were more clearly divided into rooms than was the case in LBK architecture (Tripković 2009).

As already noted, only two certain cemeteries, at Botoš and Gomolava, are known in the Vinča world, and human remains are otherwise very scarce on Vinča tells and other settlements. In contrast, a much more visible mortuary tradition is known in Transdanubia. The Early Neolithic Starčevo culture is characterised by coeval settlement and burial, as at Alsónyék (Bánffy et al. 2010). In the LBK, following its Formative phase, for example at Balatonszárszó-Kis-erdei-dűlő, burials were found in the filled pits adjacent to individual longhouses as well as further away from them (Oross and Marton 2012, pp. 259–262). There is also evidence for more regular practice: at Alsónyék the LBK burials were repeatedly uncovered in the western longpits of houses (Oross et al. in press b).

### The Ražište Style

Finally, Ražište-style pottery should be noted. This was first defined as a local variant of the early Sopot culture, being found especially in northeastern Croatia—more or less due south of Szederkény; other finds of Ražište-style pottery also exist north of the Drava on Hungarian territory, for example in the Karasica valley near Villány, just to the south of Szederkény (Marković 1985; Horváth 2006). Distinctive Ražište-style forms include slightly curving S-profiled vessels and gently biconical open bowls with slightly thickened shoulder line (thus different to sharper Vinča shapes), and recurrent decorative motifs include curvilinear incised patterns, with stab infill, set above the vessel shoulder and forming inverted arcs. Though it was previously proposed that the Ražište style was the outcome of interaction between the earliest Sopot, early Vinča and LBK spheres (Marković 1985, 1994), the difficulty is that we do not know the date of the early Sopot culture (Burić 2015); other interpretations of the emergence and position of the Ražište style are discussed below.

## Szederkény-Kukorica-dűlő

Szederkény was investigated by archaeologists of the Janus Pannonius Museum, Pécs, between 2005 and 2008 (Kovaliczky 2009). It is located in the central part of Baranya County, in the area of the southern Baranya hills (Fig. 2). The site lies on the southern and southeastern slopes of a low double ridge, 130–140 m above sea level, bounded by the Karasica stream to the west, and by the Monyoród stream to the east and the south. The excavated area was 1700 m long in a northeast–southwest direction, and covered nearly 12.5 ha. Beside the Neolithic features, many others from the Copper Age (Balaton-Lasinja and Baden cultures), Bronze Age (Encrusted Pottery culture and Urnfield culture), and La Tène and late Roman periods were also discovered.

The Neolithic settlement features were found in three clearly distinguishable groups in the eastern, central and western parts of the excavated area (Figs. 3, 4, 5). The eastern part is located on a low loess plateau, bounded to the east by a double ditch, which can also be dated to the Neolithic period. On its other side it is bounded by a depression, possibly formerly a stream, which divides the whole excavated area (Fig. 3). The central part is located on the eastern part of the plateau, which rises on the other side of this depression (Fig. 4). That is separated from the western part of the settlement by a zone approximately 150 m wide, which is free of Neolithic features. The western part of the settlement is located on the western side of the same plateau, rising above the floodplain of the Karasica stream (Fig. 5).

The architecture of the buildings found at Szederkény broadly conforms to the general architectural principles of the central European LBK. Traces of timber-framed houses, well known from the settlements of the central European LBK, were found in all three parts of the site (Figs. 3, 4, 5). Although in most cases postholes were not detected, house plans could be identified with confidence through the presence of characteristic paired elongated pits. The best preserved internal arrangement of postholes was found in the area of the central settlement cluster. In house H50, the postholes indicated a ground plan of three longitudinal rows and five cross-rows of timber uprights. The position of the two outermost rows of posts supporting the long walls could not, however, be documented for this building. The measurable length of the building was 17.75 m. Given that there was no indication of any internal division, this building might be compared with the *Kleinbau*-type structures of Modderman’s building typology (1972), although it must be noted that this structure would be unusually long within that category (Coolen 2006). The scheme of internal layout within houses dating to the Flomborn or later phases was devised for the western LBK (Modderman 1970, 1972), and subsequently adapted for earliest LBK houses in central Europe (Stäuble 2005; Lüning 2005). In contrast, the Formative and early LBK houses in Transdanubia were not sufficiently preserved to allow similar analysis (Bánffy 2004; Oross 2010).

Over the three parts of the Szederkény settlement, a total of 66 Neolithic house plans, orientated northeast–southwest, could be identified. The reconstructed house plans are arranged in smaller clusters in each part of the settlement, and show a more or less repeating layout within the clusters, where three or four buildings usually formed a row. Some relationships between the longpits can be observed. The house plans of parallel house rows can overlap at the front of the buildings, which clearly indicates successive building phases within the settlement clusters. In contrast, apart from a very few cases where longpits of adjacent houses partially overlap each other, there is no stratigraphic evidence for overlaps along the long sides of houses in the same row. Comparable layouts were recorded on LBK sites of the Tolna Sárköz region, at Tolna-Mözs (Marton and Oross 2012) and Alsónyék (Oross et al. forthcoming b).

Although the Neolithic features of the eastern part of the settlement were heavily disturbed by later activity, 30 Neolithic house plans could be identified, arranged in at least three clusters (Fig. 3). The arrangement of clusters and house rows can be best observed in the central part of the settlement, where 20 house plans were found, forming five clusters, each with one to three rows (Fig. 4). The western part of the settlement is the most seriously affected by Late Copper Age, Late Bronze Age, Roman and Migration Period activity. In this part of the site, 16 potential house plans could be tentatively reconstructed (Fig. 5).

Fifty graves were uncovered in the three settlement areas, the great majority in the eastern (25) and western (22) parts, with only three in the central part. The graves are located among the houses, and in several cases in the upper layer of the longpits. Left-crouched body positions were predominant, mostly orientated east–west and southeast–northwest. Only a few burials were accompanied by grave goods; with one exception in the west (Grave 3114), all of these were in the eastern part of the settlement. The most noteworthy is Grave 2484 (Fig. 6). This is accompanied by a black-topped vessel, a stone chisel, a *Spondylus* bracelet and a V-shaped *Spondylus* object. Although similar V-shaped *Spondylus* artefacts are known from central European LBK graves, the most obvious parallel is from Botoš-Živanićeva dolja, the cemetery of the early Vinča culture in the Vojvodina (Marinković 2010). The black-topped carinated bowl can also undoubtedly be assigned to the early Vinča culture (Schier 1996). By contrast, the individual in Grave 237, from the western longpit (Pit 219) of House 12, only a few metres away from Grave 2484, was buried with a pot which shows typical characteristics of the early LBK (Fig. 7).

**Fig. 9:**
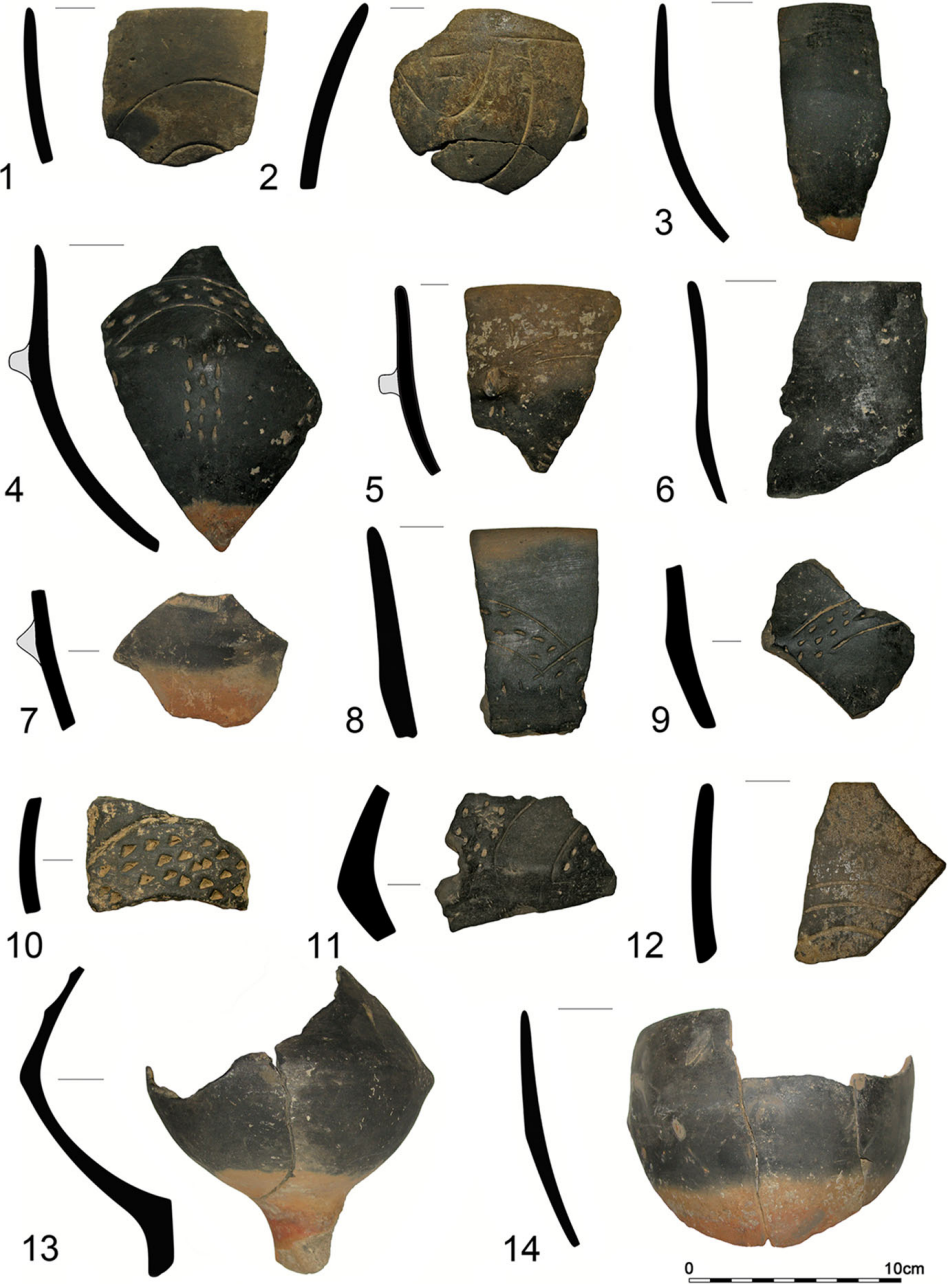
LBK-style (*1*–*2*) and Ražište-style (*3*–*14*) pottery from the western part of the settlement. *1*, *2*, *3*—House H62/Features 3350, 3379; *4*–*14*—House H51/Features 2768, 2769

**Fig. 10:**
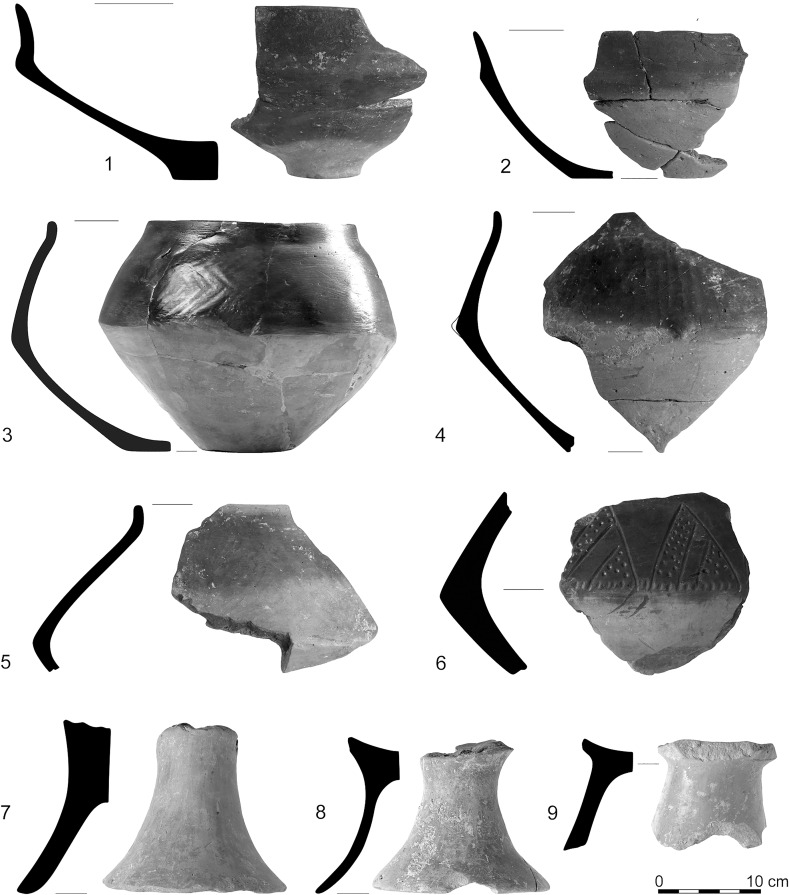
Black-topped, red-slipped vessels and red-slipped pedestals, in early Vinča style, from the eastern part of the settlement. *1*, *5*—House H4/Features 2423, 2469; *2*, *4*—House H19/Features 374, 386; *3*, *9*—House H2/Feature 31; *6*—House H22/Feature 523; *7*—House H1/Feature 55; *8*—House H12/Feature 219

The houses and their layout at Szederkény can readily be compared with central European LBK settlements, particularly with the LBK settlements of southern Transdanubia and the Balaton region. However, these houses are associated at Szederkény with material culture which is radically different to that of the LBK. The current state of post-excavation analysis indicates that the pottery assemblage of the eastern and central parts of the settlement, mainly from the elongated pits, shows strong resemblances to the early Vinča culture (Jakucs and Voicsek 2015; Figs. 8, 10). According to normal typological markers, this pottery can most probably be assigned to the A1–A3 ceramic phases of the Vinča sequence (following Schier 1996), while that from the western part of the settlement (Fig. 9) can be best associated with the Ražište style (Marković 1985; Marković and Botić 2008; Horváth 2006). There are occasional sherds of LBK types in all areas of the settlement, but these are rare (Fig. 8: 8–9; Fig. 9: 1–2), and diagnostic ceramics are overwhelmingly of the Vinča A or Ražište styles.

Preliminary typological analysis of the pottery suggested a chronology for the settlement (Jakucs and Voicsek 2015). The start was thought to involve exclusively, or almost exclusively, a group of early Vinča pottery makers (Vinča A1–A2, following Schier 1996), in the eastern and central parts of the settlement. The western part was suspected to belong to a later stage of the settlement, with a different kind of pottery that could be assigned to the Ražište style. In this part of the settlement, a somewhat larger proportion of LBK pottery was also detectable; in certain of the houses, there were some sherds that had rather late features, those of Notenkopf and the southern LBK subtype called Malo Korenovo (Težak-Gregl 1993; Tokai 2006). On the basis of all these observations, a chronological distinction was drawn between the early Vinča and the Ražište pottery styles, to the effect that the earliest activity in the Vinča subsite in the east was slightly earlier than the first appearance of the Ražište-type pottery in the western settlement cluster; it was also thought that the Ražište part was in use for longer. This was seen as in accordance with the accepted dating of Ražište-type pottery, which was thought to be coeval with Vinča A3 (Jakucs and Voicsek 2015).

Szederkény now makes possible a detailed investigation of this mix of things and practices. The focus of this paper is to produce a refined, formally modelled chronology as the first step in this investigation.

## Aims of the Szederkény Dating Programme

Szederkény was selected for inclusion in the European Research Council-funded project *The Times of Their Lives* (ToTL: see Acknowledgements) because of the mix of things and practices noted above; because of an interest in settlement histories; and because it offered comparison with the ToTL dating programme of the Vasić sequence at Vinča-Belo Brdo (Tasić et al. in press).

Specific aims of the radiocarbon dating programme were:

- to date the appearance of early Vinča-type material culture in the northernmost area of its occurrence along the Danube valley, at the southern fringes of the central European LBK
- to date the contemporary use of LBK-style longhouses and Vinča ceramics and the co-occurrence of LBK-type ceramics in overwhelmingly Vinča assemblages, thus contributing to the long-debated issue of the chronological relationship between the two cultural spheres
- to provide precise date estimates for some of the diagnostic assemblages of Vinča, Ražište, and LBK-type ceramics in the house longpits and graves
- at the site level, to determine the temporal relationships between the three parts of the settlement—east, central, and west—since (based on the material culture) there seemed to be an east to west shift through time
- and to determine whether the burials were contemporary with the nearby houses.

## Radiocarbon Dating and Chronological Modelling

The radiocarbon dating programme for Szederkény was conceived within the framework of Bayesian chronological modelling (Buck et al. 1996). Such an approach allows the combination of archaeological information with calibrated radiocarbon dates using a formal statistical methodology.

Five radiocarbon dates were obtained in 2012, from the Mannheim Radiocarbon Dating Laboratory (Tables 1, 2) as part of a joint project by the Johannes Gutenberg-Universität, Mainz, and the Archaeological Institute of the Hungarian Academy of Sciences—*Population History of the Carpathian Basin during the Neolithic and its Impact on the Peopling of Central Europe* (funded by the Deutsche Forschungsgemeinschaft)—that undertook aDNA analysis on human skeletal material from the Neolithic cultures (sixth–fifth millennium cal BC) of the Carpathian Basin (Bánffy 2013a; Szécsényi-Nagy et al. 2014, 2015). These were crouched inhumations that were dated because they had no clear Neolithic context or material culture associated with them.

**Table 1:** Szederkény-Kukorica-dűlő: radiocarbon and stable isotope results associated with the longhouse activity (Highest Posterior Density intervals are given for samples of intrinsic interest, derived from Model 1 [Fig. 11])

Laboratory number	Sample reference	Material and context	δ^13^C_IRMS_ (‰)	δ^13^C_AMS_ (‰)	δ^15^N (‰)	C/N ratio	Radiocarbon age (BP)	*Highest Posterior Density Interval (95% probability)*
**Eastern**								
MAMS-14811	Grave 2436	Human bone, rib (identified by Marc Fecher) from an adult female crouched skeleton in unfurnished Grave 2436		−22.3		3.2	6362 ± 33	*5350*–*5285* *cal BC* (*78%*) or *5275*–*5220* *cal BC* (*17%*)
OxA-29051	Grave 2484 sample A	Human bone, left femur (identified by Kitti Köhler) from a crouched skeleton of a juvenile in Grave 2484, the richest burial at the site; it was accompanied by a *Spondylus* hook, a *Spondylus* bracelet, a typical black-topped early Vinča-style bowl (A1–A2) and a stone axe	−20.0 ± 0.2		9.7 ± 0.3	3.2	6320 ± 30	*5345*–*5290* *cal BC* (*77%*) or *5270*–*5225* *cal BC* (*18%*)
SUERC-48423	Grave 2484 sample B	Replicate of OxA-29051	−19.7 ± 0.2		10.2 ± 0.3	3.2	6366 ± 34	
*Grave 2484*	*6340* *±* *23BP T′* *=* *1.0; ν* *=* *1; T′(5%)* *=* *3.8; −19.9* *±* *0.14‰ T′* *=* *1.1; ν* *=* *1; T′(5%)* *=* *3.8; 10.0* *±* *0.2‰ T′* *=* *1.4; ν* *=* *1; T′(5%)* *=* *3.8*						*6340* *±* *23*	
OxA-29050	Grave 2491	Human bone, rib (Kitti Köhler) from an adult male crouched skeleton in Grave 2491, accompanied by a *Spondylus* bracelet and a shoe-last stone adze	−19.8 ± 0.2		10.4 ± 0.3	3.3	6264 ± 34	*5310*–*5210* *cal BC*
OxA-29054	Grave 237	Human bone, left tibia (Kitti Köhler) from a crouched articulated female adult skeleton in Grave 237, accompanied by an early LBK (Bicske-Bíňa type) globular-shaped pot with incised spiraloid pattern. Cut into longpit [219] of house H12.	−20.1 ± 0.2		10.5 ± 0.3	3.2	6314 ± 33	*5295*–*5210* *cal BC*
SUERC-48425	Grave 367	Human bone, left femur (Kitti Köhler) from a crouched articulated female adult skeleton in Grave 367, accompanied by an undiagnostic spherical pot. Cut into the pit complex 364, which contained diagnostic Vinča A (A1–A3) pottery	−20.0 ± 0.2		10.1 ± 0.3	3.2	6354 ± 34	*5345*–*5280* *cal BC* (*73%*) or *5275*–*5220* *cal BC* (*22%*)
MAMS-14809	Grave 2398	Human bone, ulna from an articulated crouched adult skeleton in unfurnished Grave 2398. Cut northeastern longpit [2332] of house H27		−30.1	–	3.3	6267 ± 33	*5310*–*5210* *cal BC*
OxA-28931	Pit 186	Articulating *Bos taurus*, phalanges II–III (identified by Márta Daróczi-Szabó) from the northeastern longpit [186] of house H8, which contained a large quantity of Vinča A pottery (A1–A2). Cut by Grave 96	−18.2 ± 0.2		10.2 ± 0.3	3.3	6309 ± 32	
SUERC-48417	Pit 316	Articulating *Bos taurus*, right radius and two carpals (Márta Daróczi-Szabó) from the northwestern longpit [316] of house H16, which contained a large quantity of Vinča A pottery (A1–A2)	−22.3 ± 0.2		6.1 ± 0.3	3.2	6326 ± 34	
SUERC-48419	Pit 375—sample A	Articulating *Bos taurus*, left tibia and astragalus (Márta Daróczi-Szabó) from the northwestern longpit [375] of house H17, which contained a large quantity of Vinča A pottery. The southwestern longpit of H17 also contained a large quantity of Vinča pottery and two sherds in the Ražište style. The plans of houses H16 and H17 probably overlapped, so they could not be coeval (although their relative sequence cannot be recovered from the stratigraphic record)	−19.5 ± 0.2		9.1 ± 0.3	3.2	6247 ± 34	
OxA-28932	Pit 375—sample B	*Sus domesticus*, a pair of right metatarsals (Márta Daróczi-Szabó), from the same context as SUERC-48419	−20.5 ± 0.2		8.3 ± 0.3	3.3	6297 ± 31	
OxA-30521	Pit 522 [SZ11]—sample A	Articulating animal bone, cattle radius and ulna (identified by Jennifer Jones), from the southeastern longpit [522] of house H22, which contained a large amount of diagnostic Vinča A (A1–A2) pottery	−20.1 ± 0.2		7.8 ± 0.3	3.1	6266 ± 32	
SUERC-54933	Pit 522 [SZ11]—sample B	Replicate of OxA-30521	−20.7 ± 0.2		9.4 ± 0.3	3.4	6295 ± 37	
*Pit 522*	*6278* *±* *25BP T′* = *0.4; ν* = *1; T′(5%)* = *3.8;* −*20.4* *±* *0.14‰ T′* = *4.5; ν* = *1; T′(5%)* = *3.8; 8.6* *±* *0.2‰ T′* = *14.2; ν* = *1; T′(5%)* = *3.8*							
SUERC-54934	Pit 529 [SZ09]	Articulating animal bone, cattle metacarpals, left and right first and second phalanges, and a right third phalanx (Jennifer Jones) from the northwestern longpit [529] of house H25, which contained a large amount of Vinča A pottery and a few sherds each of Ražište and early LBK pottery	−20.2 ± 0.2		8.5 ± 0.3	3.4	6279 ± 37	
OxA-30520	Pit 530 [SZ07]	Articulating animal bone, sheep/goat, radius and ulna (Jennifer Jones), from the northwestern longpit [530] of house H28, which contained a large amount of Vinča A pottery and altar pieces	−20.1 ± 0.2		5.9 ± 0.3	3.1	6168 ± 33	
OxA-30518	Pit 2423 [SZ14]	Articulating animal bone, cattle, metacarpal, first phalanx and second phalanges (on left condyle of bone) (Jennifer Jones), from the southeastern longpit [2423] of house H4, which contained a large amount of diagnostic Vinča A pottery (A1–A2), altar fragments, anthropomorphic figurines and a few sherds of early LBK-type ceramics	−15.9 ± 0.2		10.2 ± 0.3	3.1	6239 ± 34	
OxA-30522	Pit 219 [SZ01]	Articulating animal bone, cattle, scapho-cuboid and cuneiform (Jennifer Jones), from the northwestern longpit [219] of house H12, which contained a large amount of early Vinča A (A1–A2) pottery, altar fragments and anthropomorphic figurines	−20.4 ± 0.2		7.4 ± 0.3	3.1	6295 ± 33	
SUERC-54928	Pit 219 [SZ02]	Animal bone, cattle first phalanx with refitting unfused epiphysis (Jennifer Jones), from the same context as OxA-30522	−18.6 ± 0.2		9.1 ± 0.3	3.3	6313 ± 37	
SUERC-54929	Pit 517 [SZ05]	Articulating animal bone, pig metacarpals III and IV (Jennifer Jones), from pit [517], which contained a large amount of diagnostic Vinča A (A1–A2) pottery, a human figurine and altar fragments	−20.5 ± 0.2		10.3 ± 0.3	3.4	6259 ± 37	
**Central**								
OxA-29052	Grave 1550 sample A	Human bone, left femur (Kitti Köhler) from an articulated crouched adult female skeleton in unfurnished grave 1550. The grave cuts the pit complex that included pits [1495], [1551], and [1565], which included the longpits for houses H36 and H37. This pit complex contained a large assemblage of diagnostic Vinča A pottery	−19.9 ± 0.2		9.9 ± 0.3	3.2	6273 ± 31	
OxA-29053	Grave 1550 sample A	Replicate of OxA-29052	−19.8 ± 0.2		9.9 ± 0.3	3.2	6329 ± 31	
SUERC-48424	Grave 1550 sample B	Replicate of OxA-29052	−19.7 ± 0.2		10.3 ± 0.3	3.2	6308 ± 34	
*Grave 1550*	*6303* *±* *19BP T′* = *1.7; ν* = *2; T′(5%)* = *6.0;* −*19.8* *±* *0.12‰ T′* = *2.0; ν* = *2; T′(5%)* = *6.0; 10.0* *±* *0.17‰ T′* = *1.2; ν* = *2; T′(5%)* = *6.0*						*6303* *±* *19*	*5320*–*5220* *cal BC*
OxA-28930	Pit 2125	*Bos taurus*, left metatarsal with refitting unfused epiphysis (Márta Daróczi-Szabó) from the northwestern longpit of house H40, which contained diagnostic Vinča A pottery	−19.0 ± 0.2		8.1 ± 0.3	3.3	6260 ± 32	
SUERC-54935	Pit 1396 [SZ34]	Articulating animal bone, pig metacarpals II and III (Jennifer Jones), from the western longpit [1396] of house H34, which contained Vinča A (A1–A2) pottery	−20.7 ± 0.2		9.6 ± 0.3	3.3	6299 ± 37	
SUERC-54936	Pit 1690 [SZ41]	Articulating animal bone, cattle astragalus and calcaneum (Jennifer Jones), from the northwestern longpit [1690] of house H37, which contained Vinča A pottery (the other longpit [1495] also contained one typical early LBK vessel)	−19.6 ± 0.2		6.7 ± 0.3	3.3	6272 ± 37	
OxA-30519	Pit 2057 [SZ13]—sample A	Articulating animal bone, cattle radius and ulna (Jennifer Jones) from the southeastern longpit [2057] of house H50, which contained a few sherds of diagnostic Vinča A pottery	−21.5 ± 0.2		7.7 ± 0.3	3.1	6226 ± 33	
SUERC-54937	Pit 2057 [SZ13]—sample B	Replicate of OxA-30519	−21.2 ± 0.2		7.9 ± 0.3	3.3	6322 ± 37	
*Pit 2057*	*6269 ± 25BP T′ = 3.8; ν = 1; T′(5%) = 3.8; −21.4 ± 0.14‰ T′ = 1.1; ν = 1; T′(5%) = 3.8; 7.8 ± 0.2‰ T′ = 0.2; ν = 1; T′(5%) = 3.8*							
**Western**								
MAMS-14812	Grave 2842	Human bone, rib from crouched articulated young adult skeleton from unfurnished Grave 2842, which cut pit [2768]		−17.7	–	3.3	6220 ± 29	*5295*–*5195* *cal BC*
MAMS-14810	Grave 3413	Human bone, rib from crouched articulated adult skeleton from unfurnished Grave 3413		−14.9	–	3.3	6224 ± 29	*5300*–*5200* *cal BC*
OxA-28933	Grave 3050_human	Human bone, right femur (Kitti Köhler) from crouched juvenile skeleton, part of a double burial with an adult in Grave 3050, which was accompanied by a deposit of articulated animal bones. Both skeletons were covered with large pottery fragments in the Ražište style	−19.9 ± 0.2		10.5 ± 0.3	3.3	6118 ± 31	*5210*–*5180* *cal BC*
SUERC-48418	Grave 3050_animal	*Bos taurus*, second phalanx with refitting unfused epiphysis (identified by Éva Nyerges) from the same context as OxA-28933	−19.2 ± 0.2		9.5 ± 0.3	3.3	6078 ± 34	
SUERC-54938	Pit 2768 [SZ19]	Articulating animal bone, cattle first and second phalanges (Jennifer Jones), from the northwestern longpit [2768] of house H51, which contained a large amount of diagnostic Ražište style pottery and a few sherds of LBK-type ceramics	−20.4 ± 0.2		8.5 ± 0.3	3.4	6342 ± 37	
OxA-30517	Pit 2768 [SZ18]	Articulating animal bone, cattle first and second phalanges (Jennifer Jones), from the same context as SUERC-54938	−19.1 ± 0.2		7.3 ± 0.3	3.1	6332 ± 33	
SUERC-54939	Pit 2889 [SZ22]	Articulating animal bone, cattle metatarsal and first phalanx (Jennifer Jones), from pit [2889], potentially the eastern longpit of house H53, which contained an assemblage of diagnostic Ražište style pottery and a few sherds of LBK-type ceramics	−18.9 ± 0.2		6.5 ± 0.3	3.3	6278 ± 37	
SUERC-54943	Pit 3075 [SZ38]	Articulating animal bone, cattle, first and second phalanges (Jennifer Jones), from the eastern longpit [3075] of house H57, which contained a large quantity of diagnostic Ražište pottery and a few sherds of LBK-style ceramics	−19.8 ± 0.2		8.0 ± 0.3	3.3	6224 ± 37	
OxA-30514	Pit 3075 [SZ39]	Animal bone, pig, tibia with refitting unfused epiphysis (Jennifer Jones), from the same context as SUERC-54943	−20.3 ± 0.2		8.6 ± 0.3	3.1	6350 ± 32	
OxA-30515	Pit 3075 [SZ39]	Replicate of OxA-30514	−20.3 ± 0.2		8.5 ± 0.3	3.1	6339 ± 34	
*Pit 3075*	*6345 ± 24BP T′ = 0.1; ν = 1; T′(5%) = 3.8; −20.3 ± 0.14‰ T′ = 0.0; ν = 1; T′(5%) = 3.8; 8.6 ± 0.2‰ T′ = 0.1; ν = 1; T′(5%) = 3.8*							
OxA-30516	Pit 2948 [SZ25]	Articulating animal bone, cattle metacarpal and trapezoid carpal (Jennifer Jones), from the western longpit [2948] of house H52, which contained a large amount of Ražište pottery and a few sherds of LBK-style ceramics	−19.4 ± 0.2		8.2 ± 0.3	3.1	6168 ± 33	
OxA-30513	Pit 3370 [SZ30]	Articulating animal bone, cattle second and third phalanges (Jennifer Jones), from the western longpit [3370] of house H62, which contained a large amount of Ražište pottery with a few sherds of LBK-type pottery	−19.1 ± 0.2		10.5 ± 0.3	3.2	6250 ± 32

**Table 2:** Szederkény-Kukorica-dűlő: radiocarbon and stable isotope results associated with later Neolithic and Copper Age activity

Laboratory number	Sample reference	Material and context	δ^13^C_IRMS_ (‰)	δ^13^C_AMS_ (‰)	δ^15^N (‰)	C/N ratio	Radiocarbon age (BP)	Calibrated date (95% probability)
**Later graves**								
MAMS-14808	Grave 119	Human bone, tibia from an articulated crouched adult skeleton in unfurnished Grave 119		−25.3		3.3	6079 ± 33	5210–4890 cal BC (93%) or 4870–4850 cal BC (2%)
SUERC-48426	Grave 96	Human bone, right femur (identified by Kitti Köhler) from a crouched articulated skeleton in unfurnished Grave 96	−19.9 ± 0.2		10.7 ± 0.3	3.2	5545 ± 34	4460–4340 cal BC

Results have been calibrated using the probability method (Stuiver and Reimer 1993) and IntCal 13 (Reimer et al. 2013)

### Sampling

A rigorous procedure for extracting the necessary information to build chronological models from archaeological sites has been developed (Bayliss and Bronk Ramsey 2004; Bayliss 2009), and this was used to underpin all stages of the radiocarbon dating programme for Szederkény.

The first stage in sample selection was to identify short-lived material, which was demonstrably not residual in the context from which it was recovered. The taphonomic relationship between a sample and its context is the most hazardous link in this process, since the mechanisms by which a sample came to be in its context are a matter of interpretative judgment rather than certain knowledge. Material was selected only where there was evidence that a sample had been put fresh into its context. In this respect we were fortunate in that articulating bones and re-fitting unfused epiphyses were found in reasonable numbers in the archive (cf. Bayliss et al. in press, fig. 7). This material must have been deposited in its context very soon after death or the parts would not have remained together.

Samples from articulating bones or animal bones with re-fitting epiphyses deposited in longpits associated with houses strictly provide *termini ante quos* for the construction of longhouses. It is likely, however, that the difference between the deposition of the dated animal bones and the date of house construction is relatively small, given that none of the material can have come from the upper parts of features as the top 0.5 m or more is thought to have been machined off. Samples were also preferentially chosen from features that had relatively large assemblages of distinctive Vinča-style material culture.

Samples from inhumations were selected from graves that had direct stratigraphic relationships to house longpits: for example, Grave 237 (OxA-29054) was dug into Pit 219 (SUERC-29054). Other samples were selected from inhumations with particularly rich assemblages of grave goods, such as Grave 2484 (OxA-29051 and SUERC-48425), and in other cases to provide *termini ante quos* for features with rich Vinča-style material culture, such as Grave 96 (SUERC-48426), which cuts Pit 175.

In addition, a sample (SUERC-54929) from articulating pig metacarpals III and IV from a single isolated pit (Pit 517)—not identified as a house longpit, although it was tentatively associated with house H25—was dated, as the fill contained a large amount of diagnostic Vinča-style pottery and clay altar fragments.

Stable isotope measurements (δ^13^C and δ^15^N) on human and animal bones (Tables 1, 2) indicate that the humans consumed a diet predominantly based upon temperate terrestrial C_3_ foods (Schoeninger and DeNiro 1984; Katzenberg and Krouse 1989). Radiocarbon determinations on a ‘perfect pair’ of contemporary articulated human bone (OxA-28933) and cattle bone (SUERC-48418) from Grave 3050 are statistically consistent (T′ = 0.8; ν = 1; T′(1%) = 3.8; Ward and Wilson 1978). The radiocarbon results are, therefore, unlikely to be affected by any significant reservoir effects, for example from the consumption of freshwater fish (Bayliss et al. 2004).

The C:N ratios of all bone samples indicate that preservation was sufficiently good for accurate radiocarbon dating (Masters 1987; Tuross et al. 1988).

### Results

A total of 41 radiocarbon measurements are now available from Szederkény, including 36 obtained by the ToTL Project (Tables 1, 2). These measurements are conventional radiocarbon ages (Stuiver and Polach 1977).

The five human skeletons dated at the Curt-Engelhorn-Zentrum Archäometrie, Mannheim, were prepared by gelatinisation and ultra-filtration (Brown et al. 1988), combusted in an elemental analyser, graphitised and dated by Accelerator Mass Spectrometry (AMS) (Kromer et al. 2013). Samples of human and animal bone measured at the Oxford Radiocarbon Accelerator Unit were gelatinised and ultrafiltered (Brock et al. 2010), and combusted, graphitised and dated by AMS as described by Bronk Ramsey et al. (2004). The human and animal bone samples dated at the Scottish Universities Environmental Research Centre (SUERC), East Kilbride, were gelatinised and ultrafiltered, combusted, graphitised and dated by AMS using methods described in Dunbar et al. (2016).

Replicate measurements are available on five samples. All five groups of replicate radiocarbon measurements are statistically consistent at 95% confidence (Table 1). Four of the replicate groups of δ^13^C and δ^15^N values are also statistically consistent at 95% confidence, although the values for Pit 522 [SZ11] are divergent. The replicate δ^13^C values are statistically inconsistent at 95% confidence, but consistent at 99% confidence, although the replicate δ^15^N values are statistically inconsistent at more than 99% confidence. Both values are within the range of δ^15^N values on cattle from this site and so it is not possible to determine which value is erroneous. The δ^13^C and δ^15^N values for OxA-30518 are surprisingly enriched for a sample of cattle bone. The fragment of bone dated in Oxford clearly matches the sampled location on what is unequivocally an articulating cattle foot. Following the surprising initial measurements, collagen was extracted for a second time from this bone, using the gelatinisation protocol described by Bronk Ramsey et al. (2000). The stable isotope measurements obtained were δ^13^C −16.3 ± 0.2‰, −16.3 ± 0.2‰, −16.5 ± 0.2‰ (statistically consistent with the original measurement of −15.9 ± 0.2‰; T′ = 4.7, T′5% = 7.8; ν = 3), and δ^15^N 10.6 ± 0.3‰, 10.7 ± 0.3‰, 10.2 ± 0.3‰ (statistically consistent with the original measurement of 10.2 ± 0.3‰; T′ 2.3, T′% = 7.8; ν = 3). The cause of this unexpected enrichment is thus unexplained. The replicate radiocarbon measurements have been combined by taking a weighted mean before calibration (Table 1) and inclusion in the chronological models.

All three laboratories maintain a continual programme of quality assurance procedures, in addition to participating in international inter-comparison exercises during the period when the measurements were made (Scott 2003; Scott et al. 2010).

### Chronological Modelling

**Fig. 11:**
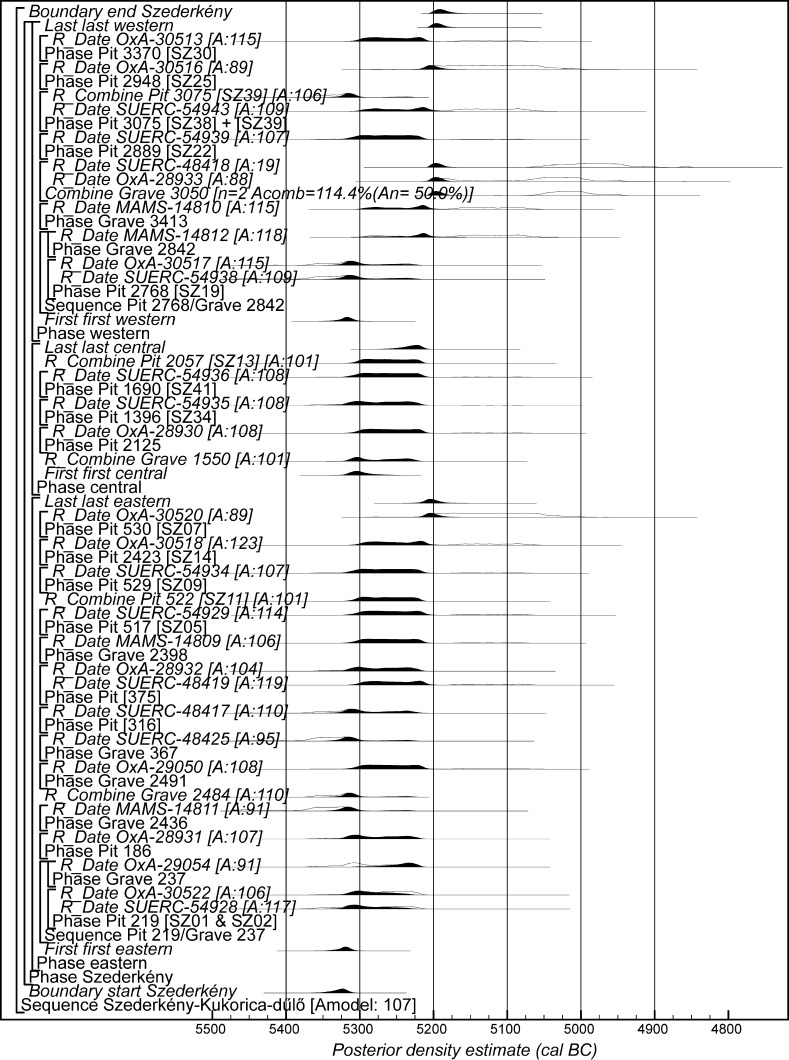
Probability distributions of radiocarbon dates from Szederkény (Model 1). Each distribution represents the relative probability that an event occurs at a particular time. For each of the dates two distributions have been plotted: one in outline, which is the result of simple radiocarbon calibration, and a solid one, based on the chronological model used. Distributions other than those relating to particular samples correspond to aspects of the model. For example, the distribution ‘*start Szederkény’* is the estimated date of the establishment of the settlement. The large square brackets down the left-hand side, along with the OxCal keywords, define the overall model exactly

Chronological modelling has been undertaken using the program OxCal v4.2 (Bronk Ramsey 2009; Bronk Ramsey and Lee 2013) and the calibration dataset of Reimer et al. (2013). The algorithms used in the models are defined exactly by the brackets and OxCal keywords on the left-hand side of Figs. 11, 14, 16, 17, 18, 19, 20, 21, 22 and 23 (http://c14.arch.ox.ac.uk/). The outputs from the models, the posterior density estimates, are shown in black, and the unconstrained calibrated radiocarbon dates are shown in outline. The other distributions correspond to aspects of the model. For example, the distribution ‘*start Szederkény’* (Fig. 11) is the posterior density estimate for the time when the settlement at Szederkény was established. In the text and tables, the Highest Posterior Density intervals of the posterior density estimates are given in *italics*.

**Fig. 12:**
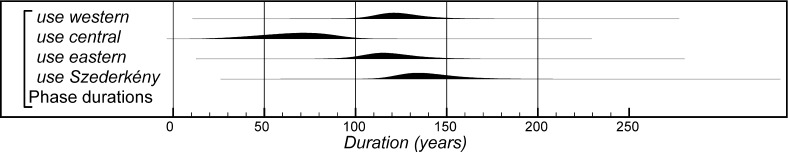
Probability distributions of durations from Szederkény (Model 1), derived from the model defined in Fig. 11

**Fig. 13:**
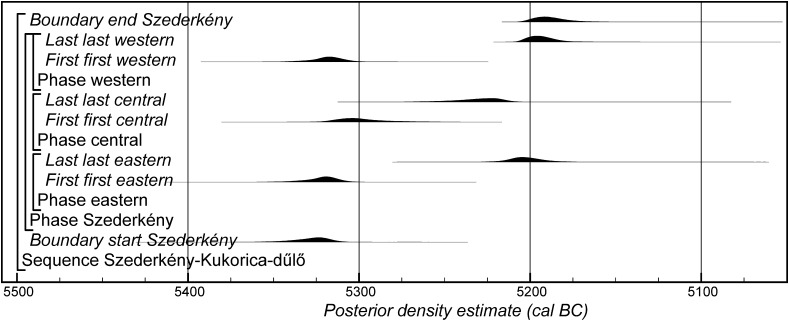
Probability distributions of key parameters from Szederkény (Model 1), derived from the model shown in Fig. 11

**Fig. 14:**
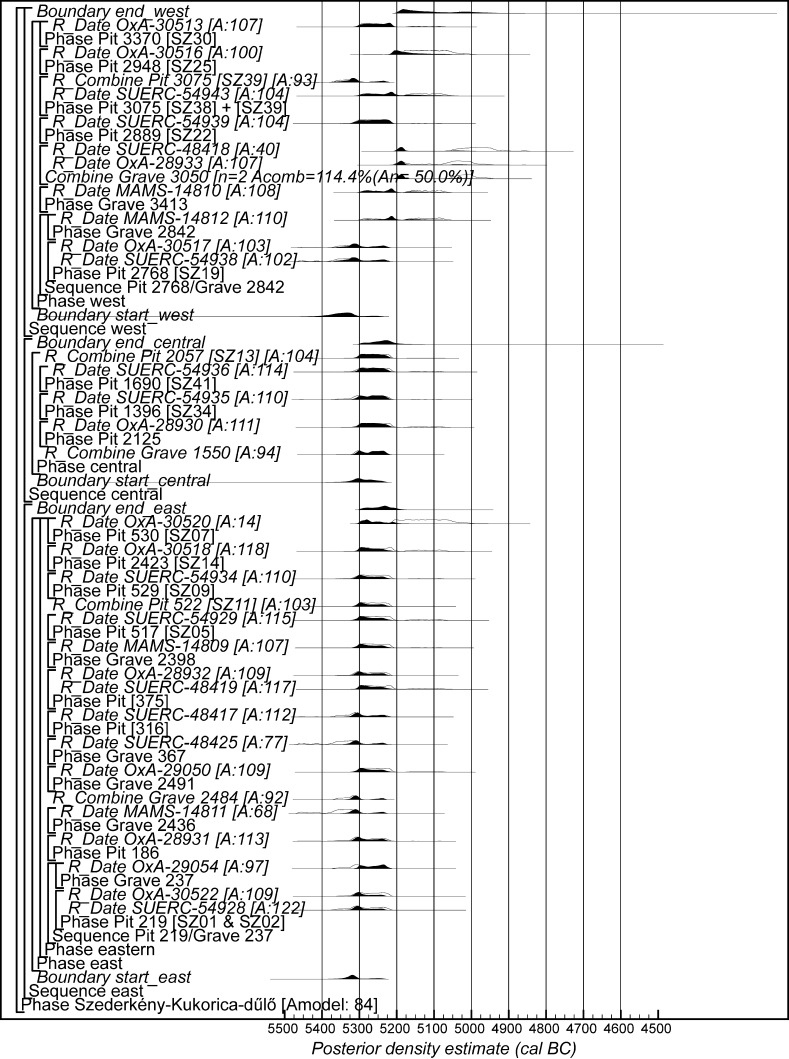
Probability distributions of radiocarbon dates from Szederkény (Model 2). The format is as Fig. 11. The large square brackets down the left-hand side, along with the OxCal keywords, define the overall model exactly

**Fig. 15:**
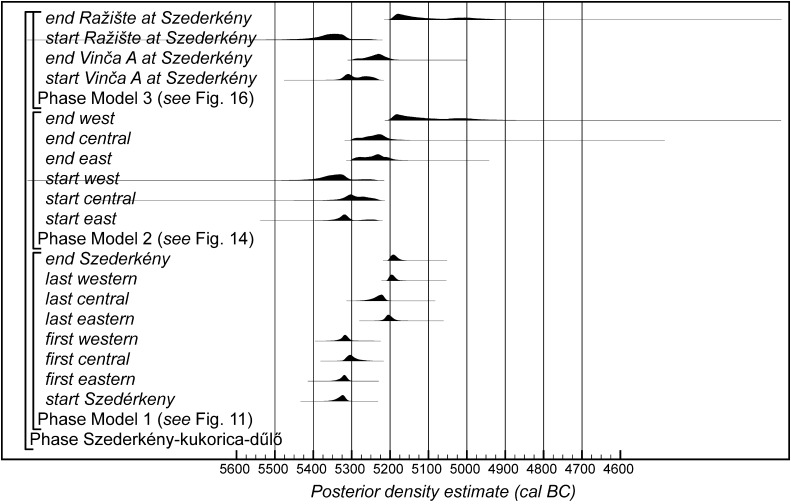
Comparison of key parameters from Szederkény, derived from the models defined in Figs. 11 (Model 1), 14 (Model 2), and 16 (Model 3)

**Fig. 16:**
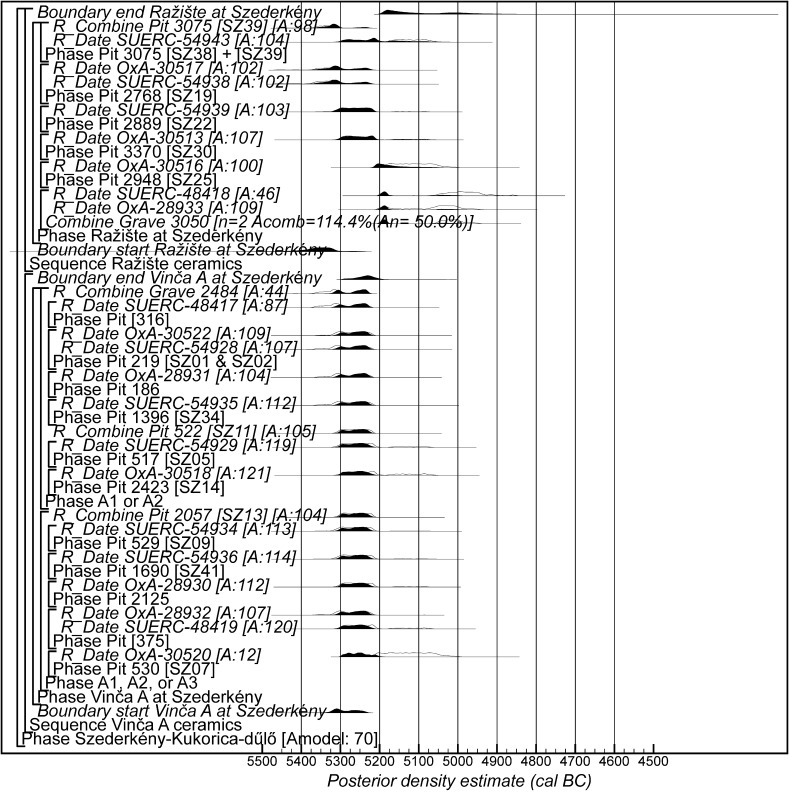
Probability distributions of radiocarbon dates from contexts directly associated with Vinča and Ražište-type ceramics at Szederkény (Model 3). The format is as Fig. 11. The large square brackets down the left-hand side, along with the OxCal keywords, define the overall model exactly

**Fig. 17:**
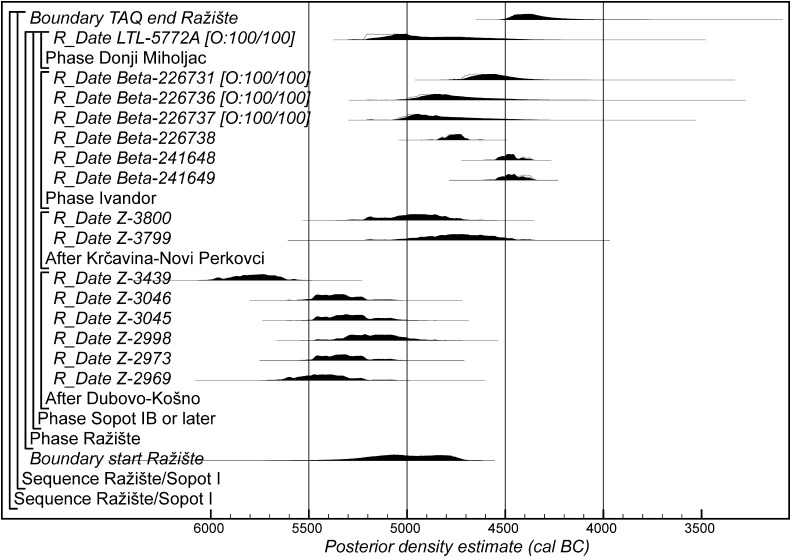
Probability distributions of radiocarbon dates from contexts directly associated with Ražište-type ceramics. The format is as Fig. 11. The large square brackets down the left-hand side, along with the OxCal keywords define the overall model exactly

**Fig. 18:**
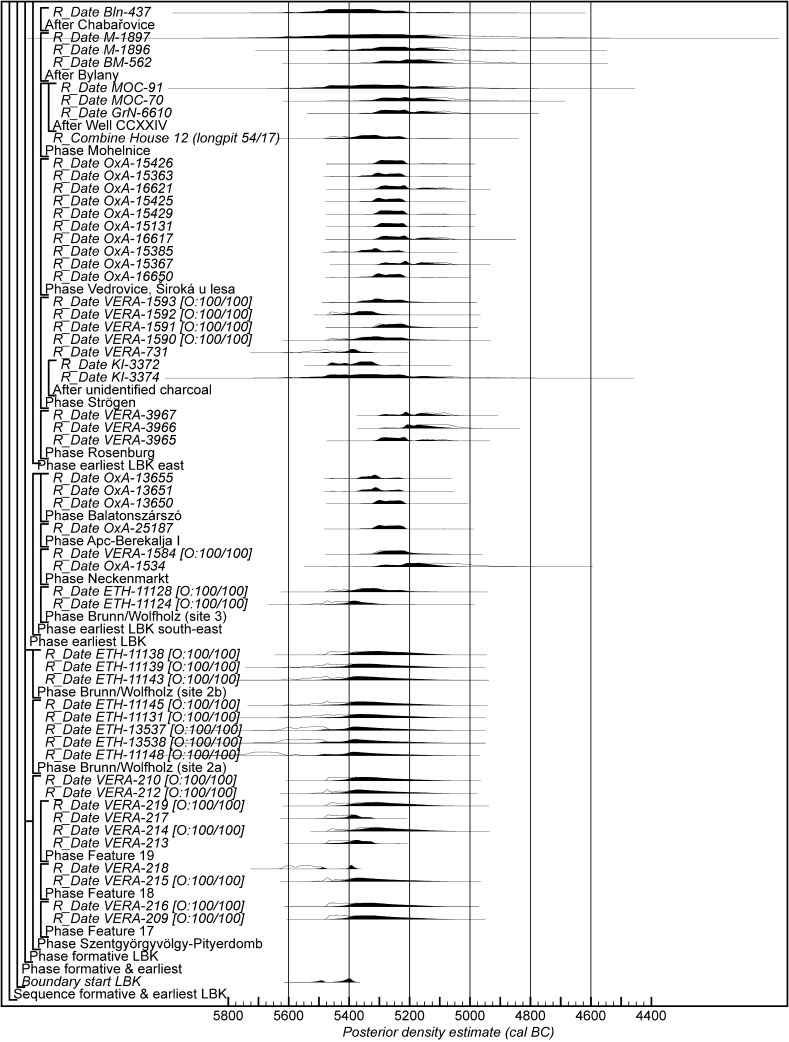
Probability distributions of radiocarbon dates from contexts directly associated with Formative and earliest LBK ceramics (LBK Model 1). The format is as Fig. 11. The large square brackets down the left-hand side of Figs. 18 and 19, along with the OxCal keywords, define the overall model exactly

**Fig. 19:**
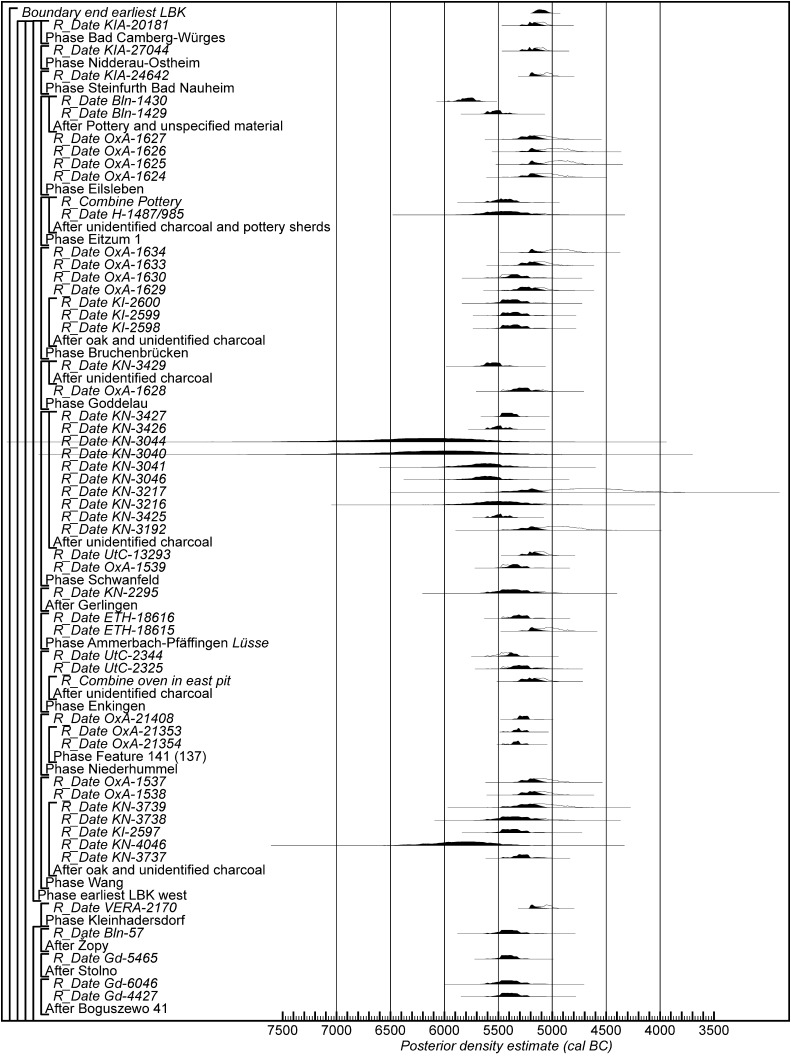
Probability distributions of radiocarbon dates from contexts directly associated with Formative and earliest LBK ceramics (LBK Model 1). The format is as Fig. 11. The large square brackets down the left-hand side of Figs. 18 and 19, along with the OxCal keywords, define the overall model exactly

**Fig. 20:**
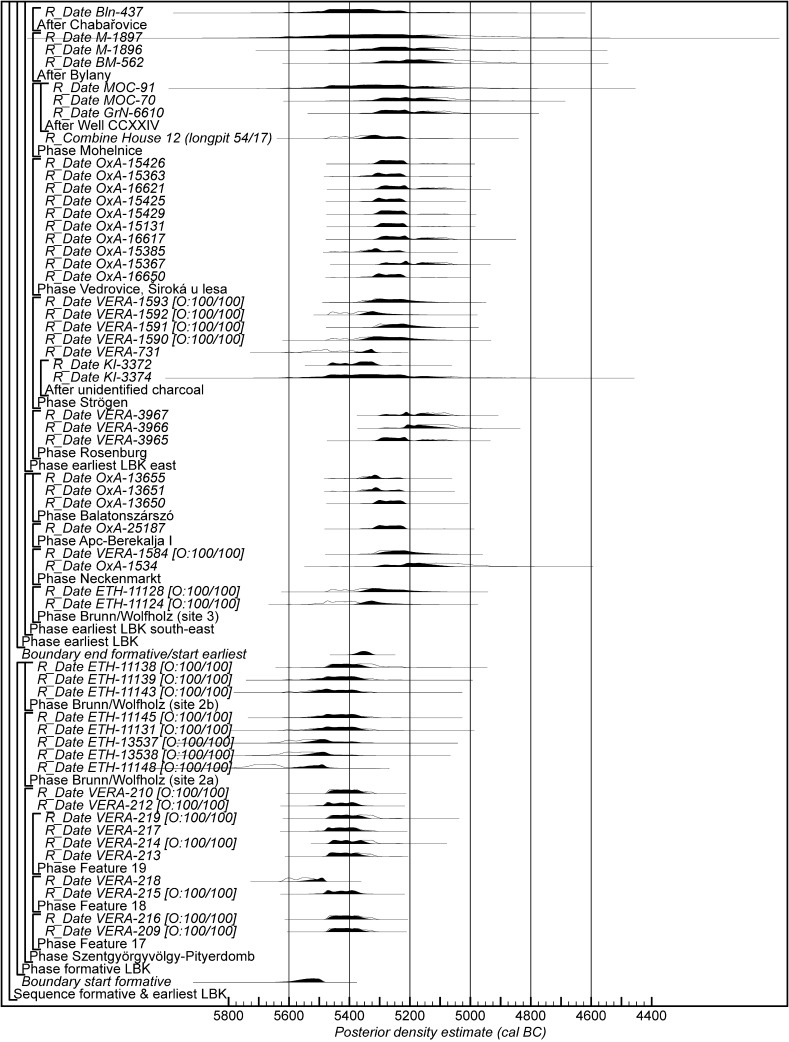
Probability distributions of radiocarbon dates from contexts directly associated with Formative and earliest LBK ceramics (LBK Model 2). The format is as Fig. 11. The large square brackets down the left-hand side of Figs. 20 and 21, along with the OxCal keywords, define the overall model exactly

**Fig. 21:**
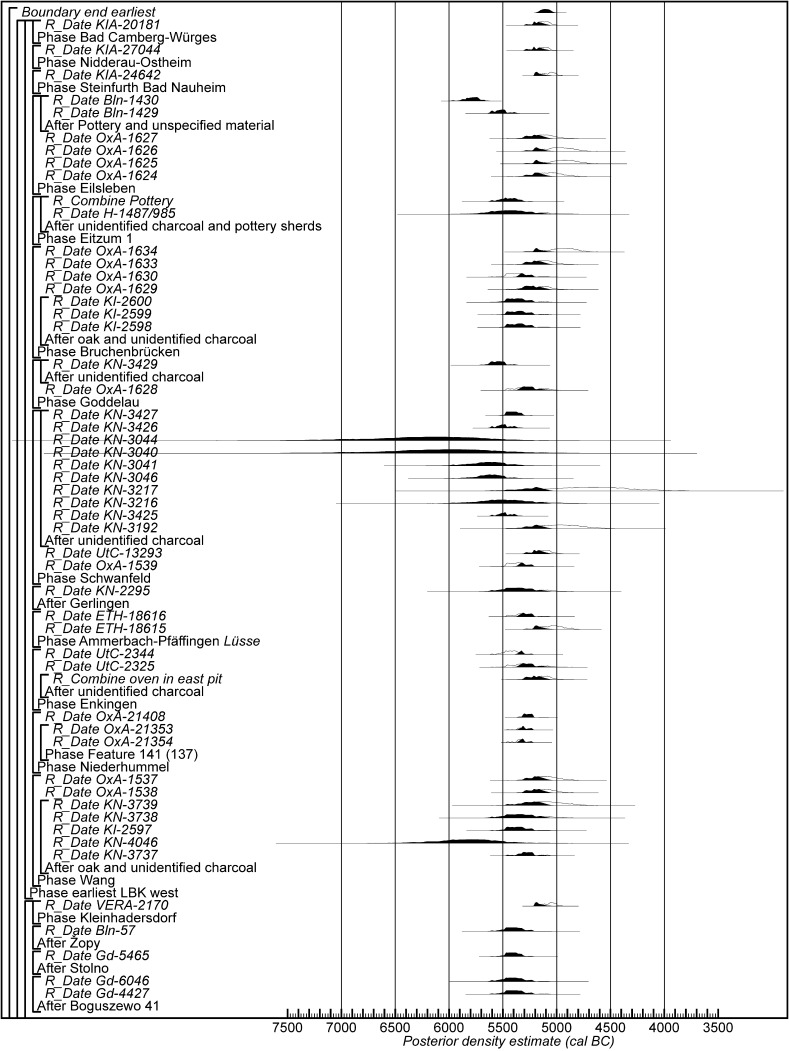
Probability distributions of radiocarbon dates from contexts directly associated with Formative and earliest LBK ceramics (LBK Model 2). The format is as Fig. 11. The large square brackets down the left-hand side of Figs. 20 and 21, along with the OxCal keywords, define the overall model exactly

**Fig. 22:**
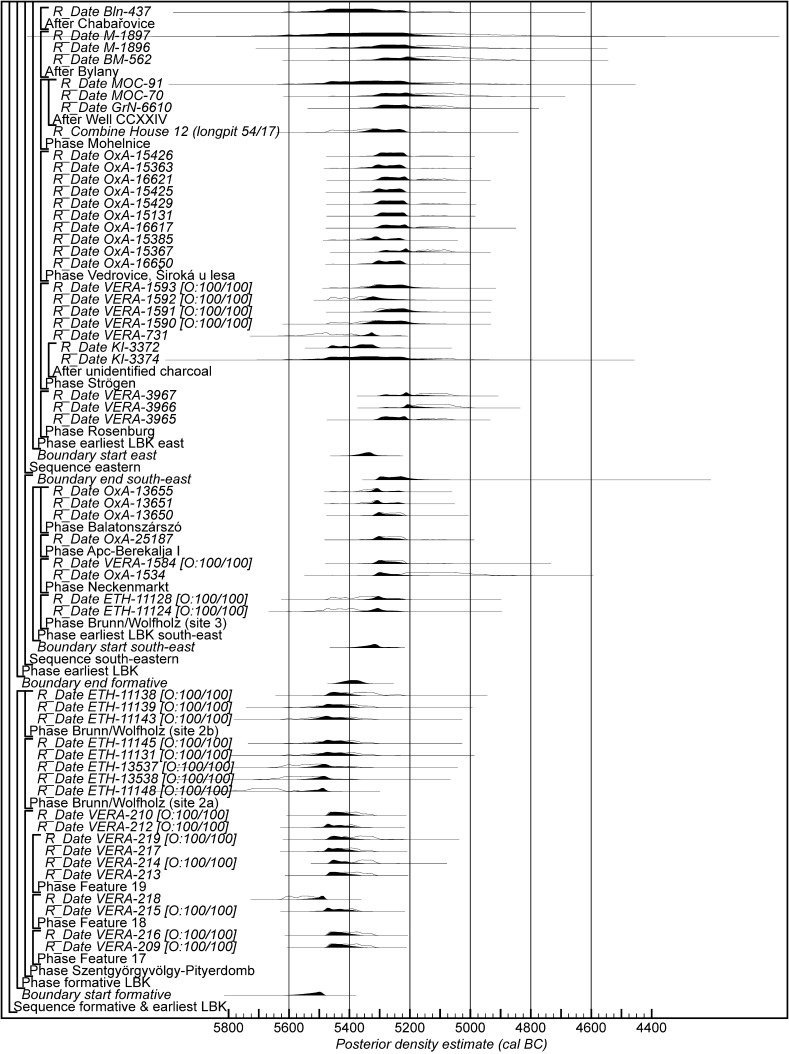
Probability distributions of radiocarbon dates from contexts directly associated with Formative and earliest LBK ceramics (LBK Model 3). The format is as Fig. 11. The large square brackets down the left-hand side of Figs. 22 and 23, along with the OxCal keywords, define the overall model exactly

**Fig. 23:**
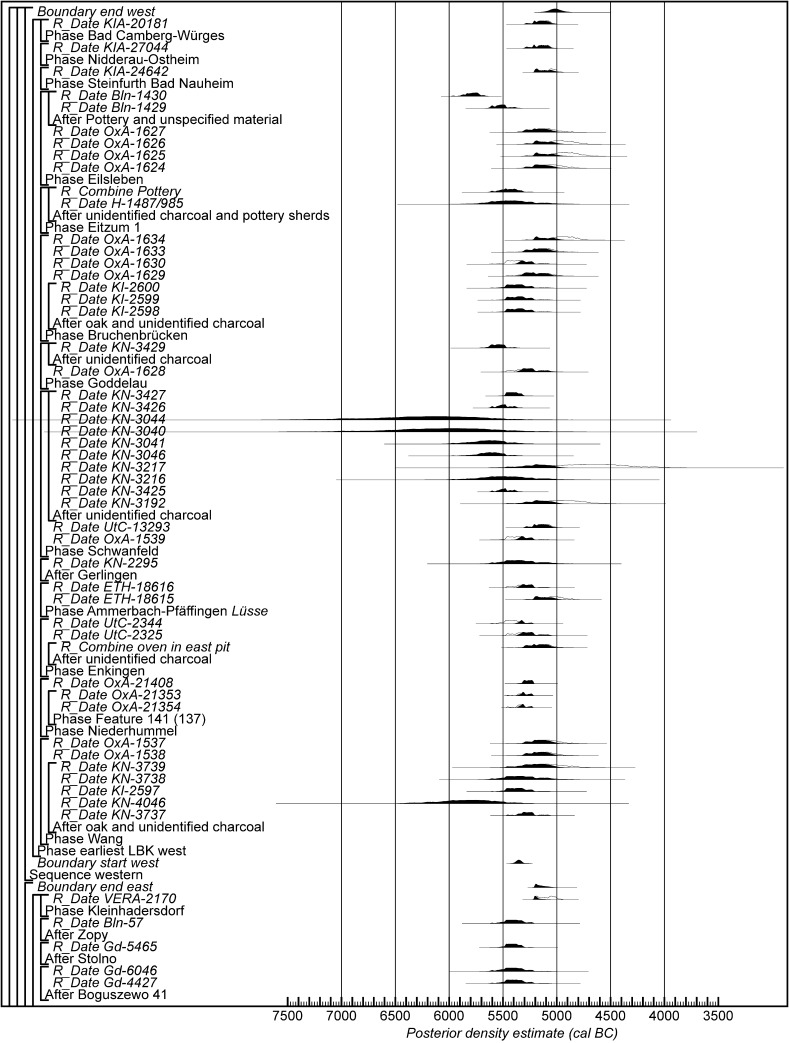
Probability distributions of radiocarbon dates from contexts directly associated with Formative and earliest LBK ceramics (LBK Model 3). The format is as Fig. 11. The large square brackets down the left-hand side of Figs. 22 and 23, along with the OxCal keywords, define the overall model exactly

A number of alternative models for understanding the chronology of Szederkény are outlined below.

#### Model 1

The first model combines the few available stratigraphic sequences for the dated samples with the radiocarbon dates in a single continuous phase of activity (Buck et al. 1992). This model assumes that the three parts of the settlement formed a coherent complex and that the occupation of the separate parts was linked.

Thirty-nine radiocarbon dates are included in this model. The radiocarbon dates from two of the unfurnished graves, Graves 96 and 119, are not included as they clearly represent later activity (MAMS-14808 and SUERC-48426; Table 2).

In the eastern part of the settlement, Grave 237 cut longpit [219] from house H12. Houses H16 and H17 were clearly not contemporary, as their plans overlap and their longpits intercut, but their relative sequence could not be reconstructed from the stratigraphic record and so cannot be included in the model. There are no direct stratigraphic relationships between the dated features in the central part of the site. In the western part, Grave 2842 cut longpit [2768] of house H51.

This model has good overall agreement (Amodel: 107), with only one measurement having poor individual agreement (SUERC-48418; A: 19).

Model 1 suggests that the Neolithic settlement began in *5360*–*5305* *cal BC* (*95% probability*; *start Szederkény*; Fig. 11), probably in *5340*–*5315* *cal BC* (*68% probability*). The settlement ended in *5210*–*5165* *cal BC* (*95% probability*; *end Szederkény*; Fig. 11), probably in the *5190s or 5180s* *cal BC* (*68% probability*). It was thus used for a period of *110*–*180* *years* (*95% probability*; *use Szederkény*; Fig. 12), probably for *120*–*155* *years* (*68% probability*).

By calculating the first and last dated events in each part of the site, we can assess their contemporaneity (Fig. 13). Occupation appears to have occurred from the beginning, in both the eastern and western parts of the settlement. It is *83% probable*, however, that the central part was first occupied a few decades later, and *97% probable* that the central part was abandoned first; it is *82% probable* that the western part of the settlement was abandoned last. The Highest Posterior Density intervals for the first and last dated events in each area of Szederkény are given in Table 3.

**Table 3:** Highest Posterior Density intervals for the first and last dated events in each area of the Szederkény longhouse site, derived from Model 1 (Fig. 11)

*Parameter*	*Highest Posterior Density interval (95% probability)*	*Highest Posterior Density interval (68% probability)*
*start Szederkény*	*5360*–*5305* *cal BC*	*5340*–*5315* *cal BC*
*end Szederkény*	*5210*–*5165* *cal BC*	*5200*–*5180* *cal BC*
*first eastern*	*5350*–*5300* *cal BC*	*5330*–*5310* *cal BC*
*last eastern*	*5230*–*5175* *cal BC*	*5215*–*5190* *cal BC*
*first central*	*5325*–*5260* *cal BC*	*5320*–*5285* *cal BC*
*last central*	*5265*–*5205* *cal BC*	*5240*–*5210* *cal BC*
*first western*	*5350*–*5290* *cal BC*	*5330*–*5305* *cal BC*
*last western*	*5210*–*5175* *cal BC*	*5205*–*5185* *cal BC*

#### Model 2

The second model recognises the differences in material culture between the areas of the site. The eastern and central areas are dominated by early Vinča-type ceramics, though they are spatially distinct. In contrast, the western area is dominated, on the basis of current evaluation, by Ražište-type pottery. Spatially, however, the western area, although separate from the central area, is less clearly divided from it.

Model 2 therefore treats activity in the three areas as independent phases of occupation—effectively as individual hamlets, a few hundred metres apart. It is essentially three models, each of which contains only the radiocarbon dates and stratigraphic information from the relevant area of the site. This means that the date estimates provided are less precise than those from Model 1, since they are based on fewer data.

This model is shown in Fig. 14 and has good overall agreement (Amodel: 84). The chronological relationships between the occupation phases of the different areas are the same as those suggested by Model 1, although the date estimates produced by Model 2 are less precise (Fig. 15). The long tails on these distributions, particularly for the western area, result from the paucity of the dates available for each area, which are insufficient entirely to constrain the scatter on the radiocarbon dates (Bayliss et al. 2007). Settlement begins more or less at the same time in the second half of the 54th century cal BC in the eastern and western areas of the site. A few decades later the central area of the site is first occupied. Again, occupation of the eastern and central areas of the site probably ends in the second half of the 53rd century cal BC, with occupation of the western area continuing into the early decades of the 52nd century cal BC.

#### Model 3

The third model combines the radiocarbon dates with the typological assessment of the associated ceramic assemblages. Two independent phases of activity are modelled, one associated with diagnostic Vinča A ceramics, and the other associated with the use of Ražište-type pottery at Szederkény. Small numbers of LBK sherds can be found in assemblages dominated by each of these types, and sometimes small numbers of Ražište-type sherds are found in assemblages that are basically Vinča A. No instances of diagnostically Vinča A sherds in Ražište-type assemblages have so far been found.

Only radiocarbon dates from features that contained diagnostic assemblages of the relevant pottery type have been included in Model 3. Nineteen radiocarbon dates from 14 features, all in the eastern and central areas of the site, are included in the model for the currency of Vinča A ceramics at Szederkény (Fig. 16). This model suggests date estimates for the use of Vinča A ceramics between the last decades of the 54th century cal BC and the latter part of the 53rd century cal BC. These are closely comparable to the estimates for the use of the eastern and central areas of the site from Models 1 and 2 (Fig. 15). Only ten radiocarbon dates from six features, all in the western area of the site, are directly associated with Ražište-type ceramics (Fig. 16). This model suggests the use of Ražište-type ceramics between the 54th and 52nd centuries cal BC at Szederkény—date estimates that are compatible with those produced for the use of the western area of the site by Models 1 and 2.

We clearly do not have sufficient radiocarbon measurements on samples directly associated with either ceramic type to counteract adequately the scatter of the radiocarbon dates.

#### Model Comparison

Figure 15 shows key parameters from all three models. These are clearly compatible, although only Model 1 includes all the information we have about the chronology of Szederkény. Since the areas of the site were clearly in contemporaneous use, the suggestion that occupation in each area was entirely unrelated seems implausible. For this reason, we prefer Model 1 as the most plausible chronology currently available for the longhouse settlement at Szederkény.

## Comparative Chronologies

### Looking South: Material Culture

The pottery identified at Szederkény clearly looks south. Wider ToTL modelling of the development of pottery in the Vinča network as a whole is under way but not yet complete. Modelled date estimates are available, however, for the ceramic typological sequence from the Vasić excavations at Vinča-Belo Brdo itself (Tasić et al. in press). The principal distribution of the Ražište style is also found to the south of Szederkény. As it now appears, the Ražište style is probably either a forerunner of the Sopot culture or its earliest manifestation. On the basis of the available evidence, the Ražište style might have come out of some kind of fusion between the earliest Vinča and the LBK ceramic traditions, preceding the emergence of the Sopot culture, or it might have been yet another outcome of the general cultural transformation affecting the whole region.

Existing dating of the Sopot I/Ražište tradition is scant (Burić 2015). Sixteen radiocarbon measurements are available from four sites that have been published as coming from contexts containing Ražište or Sopot IB–II pottery, but nine of these are conventional dates on bulk samples of unidentified charcoal, which only provide *terminipost quos* for those contexts (Table 4). Three more are apparently AMS measurements on single fragments of unidentified charcoal, which have been modelled using the Charcoal Outlier function of OxCal v4.2 (Dee and Bronk Ramsey 2014). A measurement from Donji Miholjac is on unknown material and so has also been modelled using the Charcoal Outlier function, on the precautionary principle. Three samples of bone and tooth from Ivandvor thus provide the only certainly short-lived samples in the model shown in Fig. 17. These dates simply confirm that Sopot IB–II occurs at Ivandvor in the second quarter of the fifth millennium cal BC. A 17th measurement on a human burial associated with the early phase of the Sopot culture at Kneževi-Vinogradi Osnova škola is currently inadequately published and so cannot be included in this model.

**Table 4:** Radiocarbon results associated with Ražište and early Sopot ceramics

Laboratory number	Sample reference	Material and context	Radiocarbon age (BP)	Notes and references
**Donji Miholjac, Golinci**				
LTL 5772A	No reference	Material unknown; the dated context contained pottery that was identified as Sopot I–Ražište style	6160 ± 45	Čataj and Janeš (2013), Marković (2012)
**Dubovo-Košno**				
Z-2969	152 pit SU160	Unidentified charcoal sample, Sopot IB–II	6270 ± 140	Burić (2015)
Z-2973	214 SU 148	Unidentified charcoal sample, Sopot IB–II	6350 ± 100	Burić (2015)
Z-2998	SU 1144	Unidentified charcoal sample, Sopot IB–II	6220 ± 100	Burić (2015)
Z-3045	SU 1804	Unidentified charcoal sample, Sopot IB–II	6320 ± 100	Burić (2015)
Z-3046	SU 308	Unidentified charcoal sample, Sopot IB–II	6380 ± 100	Burić (2015)
**Kneževi Vinogradi-Osnovna škola**				
Unknown	Grave	Human burial, associated with the early phase of Sopot culture	Unknown	Reported as 5480–5200 cal BC only; Šimić (2012), Burić (2015)
**Krčavina-Novi Perkovci**				
Z-3799	Pit SE619/620	Unidentified charcoal from Pit SE619/620; the published Sopot material belongs to the Ražište style	5862 ± 138	Marković and Botić (2008), Burić (2015)
Z-3800	Pit SE 621/622	Unidentified charcoal from Pit SE 621/622; the published Sopot material belongs to the Ražište style	6040 ± 100	Marković and Botić (2008), Burić (2015)
**Ivandvor**				
Beta-241649	SU 90	Tooth, from a feature containing pottery of Sopot IB–II	5620 ± 50	Burić (2015)
Beta-241648	SU 195	Bone, from a feature containing pottery of Sopot IB–II	5640 ± 40	Burić (2015)
Beta-226738	SU 41	Bone, from a feature containing pottery of Sopot IB–II	5890 ± 40	Burić (2015)
Beta-226737	SU 407	Unidentified charcoal, from a feature containing pottery of Sopot IB–II	6060 ± 40	Burić (2015)
Beta-226736	SU 407	Unidentified charcoal, from a feature containing pottery of Sopot IB–II	6000 ± 50	Burić (2015)
Beta-226731	SU 194	Unidentified charcoal, from a feature containing pottery of Sopot IB–II	5780 ± 50	Burić (2015)

If the dating of Ražište-type pottery at Szederkény must for the present stand alone, radiocarbon dates and formal modelling of the chronologies of Vinča ceramics are available (Borić 2009, 2015; Orton 2012). Here we compare the dating of Szederkény with the much studied pottery for the Vasić archive at Vinča-Belo Brdo, which has been the subject of a separate exercise in radiocarbon dating and formal modelling (Tasić et al. in press). Vinča A1 pottery appears at Szederkény in *5360*–*5305* *cal BC* (*95% probability*; *start Szederkény*; Fig. 11), probably in *5340*–*5315* *cal BC* (*68% probability*). This is clearly (*99% probable*) earlier than the appearance of the same pottery type at Belo Brdo, in *5305*–*5255* *cal BC* (*95% probability*; *start Vinča*-*Belo Brdo*; Tasić et al. in press, fig. 17), probably in *5300*–*5270* *cal BC* (*68% probability*). Occupation at Szederkény ended in *5210*–*5165* *cal BC* (*95% probability*; *end Szederkény*; Fig. 11), probably in the *5190s or 5180s* *cal BC* (*68% probability*). It is *84% probable* that this was before the transition from Vinča A3 to Vinča B1 at Belo Brdo, which occurred in *5200*–*5125* *cal BC* (*95% probability*; *Schier 4/5a*; Tasić et al. in press, fig. 22), probably in *5195*–*5155* *cal BC* (*68% probability*) (note that Tasić et al. [in press, fig. 17] show a model for Schier’s site-specific correspondence analysis for Belo Brdo [Schier 2000]; *start Vinča*-*Belo Brdo* is equivalent to the beginning of Vinča A1; *Schier 2b/3* to the transition from Vinča A1 to A2; *Schier 3/4* to A2/3; and *Schier 4/5a* to A3/B1). Vinča ceramics at Szederkény, however, were only dominant in the eastern and central parts of the settlement, which ended in *5230*–*5175* *cal BC* (*95% probability*; *last eastern*; Fig. 11), probably in *5215*–*5190* *cal BC* (*68% probability*). It is *96% probable* that this ending preceded the appearance of Vinča B1 pottery at Belo Brdo.

### Looking North: Architecture

The longhouses with flanking pits identified at Szederkény can clearly be related to those found in the LBK network to the north. As discussed above, comparable buildings have not been found in the established Vinča world to the south, although there is much uncertainty as to the range of architectural forms in the early Vinča orbit. We do not know the form of any Starčevo buildings in Transdanubia, though their presence at Alsónyék-Bátaszék is strongly suspected (Bánffy et al. 2010; Bánffy 2013b), nor do we have much information about Starčevo buildings in Croatia and Serbia. We do know of Körös houses on the Great Hungarian Plain, but these are not longhouses with flanking pits and are much less standardised—including in their orientation—than LBK structures (Raczky 2006). So we want to know about the place of the Szederkény examples within the currency of longhouses with flanking pits across their known distribution at this period. We have targeted sites with Formative and earliest (*älteste*) LBK pottery—the latter in central and western Europe—to identify sites that might be contemporary with Szederkény. We have excluded the Great Hungarian Plain and the LBK further east to make this task manageable, in the current state of research.

The data considered in this comparative exercise are listed in Table 5. Ideally, we wish to include in our models only radiocarbon dates on short-life samples that are directly associated with the relevant pottery, in this case Formative or earliest (*älteste*) LBK ceramics. Dates on human skeletons in graves containing these types of pots, for example, are ideal (for the potential of this approach, see Denaire et al. [accepted]). Unfortunately, both the quality of the samples submitted for dating by past researchers and the quality of the reporting of the resultant measurements and contextual information are inadequate (Bayliss 2015). In these circumstances, we have been forced to make pragmatic judgements about the information available to us.

**Table 5:** Radiocarbon and stable isotope results associated with Formative or earliest (*älteste*) LBK ceramics

Laboratory number	Context	Material	δ^13^C (‰)	Radiocarbon age (BP)	References
**FORMATIVE LBK**					
**Szentgyörgyvölgy-Pityerdomb**					
VERA-209	Trench II, Feature 17: round, shallow pit adjacent to House 2. Abundant finds of pottery and lithics. Pottery from the site is described as having a mixture of Starčevo and LBK traits: Formative LBK	*Quercus* sp. charcoal (all samples summarised as ‘charred twigs, branches, firewood’: Bánffy 2004, p. 299)	−26.4 ± 0.6	6420 ± 35	Bánffy (2004)
VERA-216	Trench II, Feature 17: as above	*Fagus* sp. charcoal	−26.1 ± 0.6	6420 ± 40	Bánffy (2004)
VERA-215	Trench II, Feature 18: small burnt pit interpreted as fireplace, adjacent to House 2. Abundant pottery, lithics and grinding stone	*Quercus* sp. charcoal	−29.9 ± 0.6	6475 ± 40	Bánffy (2004)
VERA-218	Trench II, Feature 18: as above	*Cornus mas* charcoal	−32.2 ± 0.6	6610 ± 40	Bánffy (2004)
VERA-213	Trench II, Feature 19: irregular, pit associated with House 2. Abundant pottery and lithics	*Fagus* sp. charcoal	−25.0 ± 0.6	6415 ± 40	Bánffy (2004)
VERA-214	Trench II, Feature 19: as above	*Ulmus* sp. charcoal	−25.4 ± 0.6	6380 ± 35	Bánffy (2004)
VERA-217	Trench II, Feature 19: as above	*Cornus mas* charcoal	−25.7 ± 0.6	6450 ± 45	Bánffy (2004)
VERA-219	Trench II, Feature 19: as above	*Fagus* sp. charcoal	−29.7 ± 0.6	6390 ± 50	Bánffy (2004)
VERA-212	Trench I, Feature 9: long pit on east side of House 1. Abundant pottery, lithics and a grinding stone	*Fagus* sp. charcoal	−25.2 ± 0.6	6475 ± 40	Bánffy (2004)
VERA-210	Trench II, Feature 21: elongated pit associated with House 2. Abundant pottery, some lithics, grinding stone and whetstone	*Quercus* sp. charcoal	−25.5 ± 0.6	6425 ± 35	Bánffy (2004)
**Brunn/Wolfholz site 2a**					
ETH-11148	Obj. 0721: 6144	Unidentified charcoal		6785 ± 75	Lenneis and Stadler (1995), cf. Stadler and Kotova (2010)
ETH-13538	Obj. 1216 E: 10063	Unidentified charcoal		6605 ± 85	Lenneis and Stadler (1995), cf. Stadler and Kotova (2010)
ETH-13537	Obj. 1202 B: 10026	Unidentified charcoal		6565 ± 85	Lenneis and Stadler (1995), cf. Stadler and Kotova (2010)
ETH-11131	Obj. 0114 O1: 01061e	Unidentified charcoal		6485 ± 80	Lenneis and Stadler (1995), cf. Stadler and Kotova (2010)
ETH-11145	Obj. 1000P5/6: 06083	Unidentified charcoal		6480 ± 70	Lenneis and Stadler (1995), cf. Stadler and Kotova (2010)
**Brunn/Wolfholz site 2b**					
ETH-11143	Obj. 0180C: 1388	Unidentified charcoal		6505 ± 75	Lenneis and Stadler (1995), cf. Stadler and Kotova (2010)
ETH-11139	Obj. 0149A: 1383	Unidentified charcoal		6470 ± 75	Lenneis and Stadler (1995), cf. Stadler and Kotova (2010)
ETH-11138	Obj. 0145	Unidentified charcoal		6390 ± 65	Lenneis *et al*. (1996), cf. Stadler and Kotova (2010)
**EARLIEST LBK**					
***Southeast group***					
**Brunn/Wolfholz site 3**					
ETH-11124	Site 3. Obj.0051	Unidentified charcoal		6470 ± 55	Lenneis and Stadler (1995)
ETH-11128	Site 3. Obj.0051	Unidentified charcoal		6360 ± 60	Lenneis and Stadler (1995)
**Neckenmarkt**					
OxA-1534	Grube 1, Teil D, Q.121: Stratum f. Pit associated with site phase 1 (assigned to the ‘late early phase of the earlier Linear Pottery culture’: Lenneis and Lüning 2001, p. 223)	Carbonised cereal	−26.0	6170 ± 80	Whittle (1990), Lenneis and Lüning (2001)
VERA-1584	Pit 113, Stratum c: 113–34: pottery from Pit 113 assigned to first site phase, early phase 1a of earlier LBK (Lenneis and Lüning 2001, p. 164)	Unidentified charcoal		6280 ± 40	Lenneis and Lüning (2001), Lenneis and Stadler (2002)
**Balatonszárszó**					
OxA-13650	Grave 792. Left-crouched, ENE–WSW oriented body of a 40–59-year-old male. No grave goods uncovered. On a surface with features containing earliest/*älteste* LBK material culture, in Pit B-4969	Human bone	−19.5 ± 0.2	6292 ± 33	Krisztián Oross, *pers. comm.*
OxA-13651	Grave 793. Left-crouched, ESE–WNW oriented body of a 23–39-year-old male. No grave goods uncovered. NE area of the excavation. On a surface with earliest/*älteste* LBK material culture	Human bone	−19.6 ± 0.2	6330 ± 33	Krisztián Oross, *pers. comm.*
OxA-13655	Settlement pit (5686) with typical Bicske-Bíňa type LBK pottery. On a surface with earliest/*älteste* LBK material culture	Disarticulated cattle bone	−21.3 ± 0.2	6339 ± 32	Krisztián Oross, *pers. comm.*
**Apc-Berekalja I**					
OxA-25187	Pit 697, SW section, 2nd spit. Associated with pottery of earliest LBK style	Disarticulated cattle cortex	−19.9 ± 0.2	6290 ± 40	László Domboróczki and Alasdair Whittle, *pers. comm*.
***East group***					
**Rosenburg**					
VERA-3965	House 1, flanking Grube 1, Pos-Nr 1. Houses 1–3 belong to site phase 1, oldest LBK, equivalent to Moravian 1b (Lenneis 2009, 81)	Animal bone		6245 ± 40	Lenneis (2009)
VERA-3966	House 1, flanking Grube 1, Pos-Nr 10: as above	Animal bone		6180 ± 40	Lenneis (2009)
VERA-3967	House 1, flanking Grube 1, Pos-Nr 25: as above	Animal bone		6210 ± 35	Lenneis (2009)
**Strögen**					
KI-3374	Pit 5, Q.3: Stratum 7; 5–148: small pit near House 2 belonging according to pot typology (Lenneis and Lüning 2001, 223; Lenneis 2009) to the second part of regional phase 1a	Unidentified charcoal		6350 ± 140	Lenneis and Lüning (2001)
KI-3372	Pit 5, Q.3: Stratum 10; 5–160: as above	Unidentified charcoal		6380 ± 140	Lenneis and Lüning (2001)
VERA-731	Pit 5, Q.4, Stratum 5; 5–71: as above	Carbonised cereal	−28.1 ± 1.6	6510 ± 60	Lenneis and Lüning (2001), Lenneis and Stadler (2002)
VERA-1590	Pit 5, Stratum 6; 5–87: as above	*Fraxinus* sp. charcoal		6340 ± 60	Lenneis and Lüning (2001), Lenneis and Stadler (2002)
VERA-1591	Pit 5, Stratum 10; 5–161: as above	*Fraxinus* sp. charcoal		6285 ± 35	Lenneis and Lüning (2001), Lenneis and Stadler (2002)
VERA-1592	Pit 6, Stratum 7; 6–90: larger pit, probable flanking pit of House 3 (site phase 2), which might have some later admixture, but perhaps pottery slightly later than that in Pit 5 (Lenneis and Lüning 2001, p. 174)	*Quercus* sp. charcoal		6395 ± 30	Lenneis and Lüning (2001), Lenneis and Stadler (2002)
VERA-1593	Pit 10, Stratum 4; 10–33: larger pit, probable other flanking pit of House 3, though its earliest-style pottery not markedly diagnostic (Lenneis and Lüning 2001, p. 174)	*Quercus* sp. charcoal		6325 ± 40	Lenneis and Lüning (2001), Lenneis and Stadler (2002)
**Kleinhadersdorf**					
VERA-2170	Grave 69. Left-crouched mature male, with grinding tablet and broken pot, of Moravian phase 1B style, transition from earliest to early LBK	Human bone	−19.5	6135 ± 35	Neugebauer-Maresch and Lenneis (2015)
**Vedrovice, Široká u lesa**					
OxA-16650	Grave 15/75. Left-crouched adult male, with polished stone, pot, *Spondylus* beads and grinding stone. Moravian phase 1B1. Grave phasings by Podborský 2002	Human bone	−18.8 ± 0.2	6299 ± 35	Pettitt and Hedges (2008)
OxA-15367	Grave 30/76. Left-crouched juvenile, with polished stone, pot and ochre. Phase 1B1	Human bone	−18.7 ± 0.2	6219 ± 35	Pettitt and Hedges (2008)
OxA-15385	Grave 37/76. Left-crouched juvenile, with polished stone. Phase 1B1	Human bone	−18.9 ± 0.2	6332 ± 37	Pettitt and Hedges (2008)
OxA-16617	Grave 54/78. Left-crouched adult male, with polished stone, pot and *Spondylus* beads. Phase 1B1	Human bone	−18.9 ± 0.2	6240 ± 45	Pettitt and Hedges (2008)
OxA-15131	Grave 62/78. Left-crouched adult female, with pot and *Spondylus* bead. Phase 1B1	Human bone	−19.2 ± 0.2	6266 ± 36	Pettitt and Hedges (2008)
OxA-15429	Grave 72/79. Left-crouched adult female, with pots, shells and ochre. Phase 1B	Human bone	−18.5 ± 0.2	6268 ± 37	Pettitt and Hedges (2008)
OxA-15425	Grave 77/79. Left-crouched adult male, with polished stone, pot and worked antler. Phase 1B2	Human bone	−18.6 ± 0.2	6298 ± 34	Pettitt and Hedges (2008)
OxA-16621	Grave 79/79. Left-crouched adult male, with polished stone, pot, *Spondylus* bead, lithics and bone artefact. Phase 1B1	Human bone	−19.2 ± 0.2	6244 ± 40	Pettitt and Hedges (2008)
OxA-15363	Grave 91/80. Left-crouched young adult female, with pots and *Spondylus* beads. Phase 1B1	Human bone	−19.1 ± 0.2	6305 ± 40	Pettitt and Hedges (2008)
OxA-15426	Grave 99/81. Left-crouched adult male, with pot. Phase 1B	Human bone	−19.4 ± 0.2	6272 ± 37	Pettitt and Hedges (2008)
**Mohelnice**					
Bln-102	House 12, depth of 60 cm in east flanking pit, 54/17. Assigned to earliest LBK on style of house and absence of music-note motifs on the pottery	Carbonised cereal (*Triticum dicoccum*)		6285 ± 100	Tichý (1963, 16), Kohl and Quitta (1964, 315), ‘without chemical treatment’, Stäuble (2005), Schmidt and Gruhle (2003)
Bln-102A	House 12, depth of 60 cm in east flanking pit, 54/17. Assigned to earliest LBK on style of house and absence of music-note motifs on the pottery	Carbonised cereal (*Triticum dicoccum*)		6405 ± 100	Tichý (1963, 16), Kohl and Quitta (1964, 315), with ‘usual acid and alkali soaking’; Stäuble (2005)
GrN-6610	Well, CCXXIV	Waterlogged wood		6240 ± 65	Breunig (1987, 123), Stäuble (2005)
MOC-70	Well, CCXXIV	Waterlogged wood		6220 ± 80	Neustupný and Vesely (1977, 185), Stäuble 2005
MOC-91	Well, CCXXIV	Waterlogged wood		6330 ± 140	Neustupný and Vesely (1977, 185), Stäuble (2005)
**Žopy**					
Bln-57	‘Dwelling pit, ca. 5 m long and sunk to 1 m depth’. In upper part, below 40 cm of ‘humus soil’	Sherds, with ‘thick walls, organic temper’, and vessel forms and decoration characteristic of earliest LBK		6430 ± 100	Kohl and Quitta (1964, 315)
**Bylany**					
BM-562	‘Stelle’, period 1, 2214	Unidentified charcoal		6184 ± 89	Breunig (1987, 123), Stäuble (2005)
M-1896	Oven, period 1c	Unidentified charcoal		6250 ± 100	Pavlů and Zápotocká (1979, 302), Stäuble (2005)
M-1897	Pit, period 1c	Unidentified charcoal		6320 ± 230	Pavlů and Zápotocká (1979, 302), Stäuble (2005)
**Chabařovice**					
Bln-437	Pit	Unidentified charcoal		6400 ± 120	Breunig (1987, 124), Stäuble (2005)
**Boguszewo 41**					
Gd-4427	Pit st. 41/ob. 3	Unidentified charcoal		6420 ± 100	Jankowska (1990, 61), Stäuble (2005), Pyzel (2006), Dębiec and Saile (2015)
Gd-6046	Pit st. 41/ob. 5	Unidentified charcoal		6440 ± 120	Jankowska (1990, 61), Stäuble (2005), Pyzel (2006)
**Stolno**					
Gd-5465	?Pit st. 2/ob. 2	Unidentified charcoal		6440 ± 70	Jankowska (1990, 61), Stäuble (2005)
***West group***					
**Wang**					
KN-3737	House 1, east flanking pit, quadrant 4, Stratum 4; 22–15/92.327	*Quercus* sp. charcoal		6300 ± 65	Stäuble (2005)
KN-4046	House 1, east flanking pit, quadrant 12–16, Stratum 2-4; 22–33/77.176	*Quercus* sp. and unidentified charcoal		6900 ± 300	Stäuble (2005)
OxA-1538	House 1, east flanking pit, quadrant 46, Stratum 6; 22–108/541/564	Carbonised cereal	−26.0	6190 ± 80	Stäuble (2005)
KI-2597	House 20, west flanking pit, quadrant 4/5, Stratum 3; 42–180	Unidentified charcoal	−25.2	6390 ± 100	Stäuble (2005)
OxA-1537	House 20, west flanking pit, quadrant 8, Stratum 5; 42–127	Carbonised cereal	−26.0	6170 ± 90	Stäuble (2005)
KN-3738	Oven in pit complex, quadrant 1/4/5, Stratum 6/5/5; 58–49/60/75	*Quercus* sp. charcoal		6370 ± 160	Stäuble (2005)
KN-3739	Oven in pit complex, quadrant 4, Stratum 8; 58–81/94..144	*Quercus* sp., *Corylus* sp. and unidentified charcoal		6190 ± 150	Stäuble (2005)
**Niederhummel**					
OxA-21354	Pit, feature 141, context 137, associated with earliest LBK pottery	Carbonised cereal, indeterminate wheat grain	−25.2 ± 0.2	6347 ± 39	Hofmann and Whittle (2011)
OxA-21353	Pit, feature 141, context 137, as above	Carbonised cereal grain, possibly *Triticum monococcum*	−25.8 ± 0.2	6330 ± 38	Hofmann and Whittle (2011)
OxA-21408	Pit, feature 142, context 113, associated with earliest LBK pottery	Carbonised cereal, indeterminate wheat grain	−24.4 ± 0.2	6292 ± 39	Hofmann and Whittle (2011)
**Enkingen**					
KI-3373	Oven in east pit, quadrant 5, Stratum 6; 57–92	Unidentified charcoal		5990 ± 210	Stäuble (2005), Albert and Schröter (1971)
KI-3375	Oven in east pit, quadrant 5, Stratum 6; 57–91	Unidentified charcoal		6280 ± 140	Stäuble (2005)
KI-3376	Oven in east pit, quadrant 5, Stratum 6; 57–94	Unidentified charcoal		6212 ± 80	Stäuble (2005)
UtC-2325	House 1, west flanking pit, quadrant 41, Stratum 1; 30–252	Organic crust on pot 315, residual fraction	−26.9	6320 ± 90	Stäuble (2005)
UtC-2346	House 1, west flanking pit, quadrant 41, Stratum 1; 30–252	Organic crust on pot 315, soluble fraction	−26.0	1650 ± 80	Stäuble (2005)
UtC-2344	House 1, west flanking pit, quadrant 45, Stratum 1; 30–55	Organic crust on pot 11, residual fraction	−26.8	6460 ± 80	Stäuble (2005)
UtC-2345	House 1, west flanking pit, quadrant 45, Stratum 1; 30–55	Organic crust on pot 11, soluble fraction	−26.0	2580 ± 120	Stäuble (2005)
**Rottenburg-Fröbelweg**					
ETH-623	Base of pit, at southwest end of House A	Bone		6230 ± 90	Reim (1994), Bofinger (2005), Stäuble (2005)
ETH-9548	Pit 23, Context 3, 4	Animal bone	−22.8 ± 1.0	6230 ± 90	Reim (1994), Bofinger (2005), Stäuble (2005)
ETH-9549	Pit 96, Context 91	Animal bone	−19.2 ± 1.2	6060 ± 70	Reim (1994), Bofinger (2005), Stäuble (2005)
ETH-15741	Posthole 3, Context 7	Animal bone	−20.8 ± 1.2	5870 ± 80	Bofinger (2005)
ETH-15742	Pit 128, Context 63	Animal bone	−21.2 ± 1.2	5890 ± 75	Bofinger (2005)
ETH-15743	Pit 141, Context 81	Animal bone	−19.1 ± 1.2	5930 ± 75	Bofinger (2005)
ETH-15744	Flanking house pit 157, Context 111	Animal bone	−18.7 ± 1.2	5895 ± 80	Bofinger (2005)
ETH-15745	Pit 195, Context 60	Animal bone	−20.6 ± 1.2	5810 ± 80	Bofinger (2005)
ETH-15746	Flanking house pit 285, Context 64	Animal bone	−20.8 ± 1.2	6015 ± 75	Bofinger (2005)
ETH-15747	Flanking house pit 282, Context 84	Animal bone	−20.4 ± 1.2	6185 ± 75	Bofinger (2005)
ETH-15748	Pit 356, Context 77	Animal bone	−21.4 ± 1.2	5870 ± 75	Bofinger (2005)
ETH-15749	Pit complex 353, Context 87	Animal bone	−20.9 ± 1.2	5915 ± 80	Bofinger (2005)
ETH-15750	Pit house (*Grubenhaus*) 332, Context 65	Animal bone	−22.0 ± 1.0	5915 ± 80	Bofinger (2005)
ETH-15751	Pit 23, Context 4	Carbonised cereals	−23.1 ± 1.2	6210 ± 70	Bofinger (2005)
ETH-15752	Pit 77, Context 122	Carbonised cereals	−22.9 ± 1.2	6120 ± 70	Bofinger (2005)
**Ammerbach-Pfäffingen** ***Lüsse***					
ETH-18615	Pit	Animal bone	−21.0 ± 1.2	6115 ± 70	Albert and Schröter (1971), Bofinger (2005)
ETH-18616	Pit	Animal bone	−20.7 ± 1.2	6325 ± 70	Albert and Schröter (1971), Bofinger (2005)
**Gerlingen**					
KN-2295	Pit	Unidentified charcoal		6390 ± 160	Stäuble (2005)
**Schwanfeld**					
OxA-1539	House 6, east pit, quadrant d, Stratum 6; 127–140	Carbonised *Triticum* spikelet	−26.0	6380 ± 80	Stäuble (1995, 2005)
Hd-14394	House 8, west flanking pit, quadrant c, Stratum 3; 494-147	Left proximal radius, *Bos primigenius*	−23.1	5820 ± 45	Stäuble (1995, 2005)
Hd-14111	House 8, west part of flanking pit, quadrant d, all layers; 493-18	Female bovid, fragment of pelvis	−22.5	6343 ± 42	Stäuble (1995, 2005)
KN-3425	House 11, east flanking pit, quadrant e, Stratum 1; 353-73	Unidentified charcoal		6520 ± 64	Stäuble (1995, 2005)
KN-3216	House 11, west flanking pit, quadrant a, Stratum 5; 455-26	Unidentified charcoal		6540 ± 260	Stäuble (1995, 2005)
KN-3217	House 11, east flanking pit, quadrant a, layer uncertain; 492-19	Unidentified charcoal		5800 ± 320	Stäuble (1995, 2005)
KN-3046	House 11, post-row, quadrant b, Stratum 5; 362-12	Unidentified charcoal		6690 ± 140	Stäuble (1995, 2005)
KN-3041/2/3	House 11, post-row, quadrant b, Stratum 1-6; 255-6/7	Unidentified charcoal		6700 ± 190	Stäuble (1995, 2005)
KN-3040	House 11, post-row, quadrant b, Stratum 4-7; 254-8	Unidentified charcoal		7100 ± 500	Stäuble (1995, 2005)
KN-3044/45	House 11, post-row, quadrant a/b, Stratum 1-8; 362-6/11	Unidentified charcoal		7250 ± 500	Stäuble (1995, 2005)
KN-3192	House 11, west flanking pit, quadrant t/u/y; 360-16/74/78	Unidentified charcoal		6060 ± 170	Stäuble (1995, 2005)
KN-3426	House 12, west flanking pit, quadrant e, from ‘profile’; 562-160	Unidentified charcoal		6530 ± 70	Stäuble (1995, 2005)
KN-3427	House 12, west flanking pit, quadrant n, Stratum 2; 564-157	Unidentified charcoal		6430 ± 60	Stäuble (1995, 2005)
Hd-14219	House 16, west flanking pit, quadrant b, Stratum 16; 704/760-138	Femur from crouched burial of a man	−21.1	6580 ± 20	Stäuble (1995, 2005)
Hd-14031	House 16, west flanking pit, quadrant g, Stratum 7; 704/760-20	Red deer (*Cervus elaphus*) scapula	−22.1	6380 ± 100	Stäuble (1995, 2005)
Hd-14177	House 16, west flanking pit, quadrant o, Stratum 6; 704-387	Left proximal radius, aurochs (*Bos primigenius*)	−23.2	5785 ± 45	Stäuble (1995, 2005)
Hd-14032	House 18, east flanking pit, quadrant m, Stratum 6; 795-134	Bovid right *os centrotarsale*	−22.4	6240 ± 55	Stäuble (1995, 2005)
UtC-13293	House 19, west flanking pit, recut 796/797, Quadrant P1, level 0 to e	Human bone (child)	−22.4	6190 ± 50	Lüning (2011)
UtC-2340	House 18, west flanking pit, quadrant a, Stratum 4; 792-86	Organic crust on pot 2195, residual fraction	−26.4	6350 ± 80	Stäuble (1995)
UtC-2341	House 18, west flanking pit, quadrant a, Stratum 4; 792-86	Organic crust on pot 2195, soluble fraction	−26.0	5190 ± 90	Stäuble (2005)
UtC-2320	House 18, west flanking pit, quadrant a, Stratum 3; 792-81	Organic crust on pot 2153, residual fraction	−26.0	7900 ± 80	Stäuble (2005)
UtC-2339	House 18, west flanking pit, quadrant a, Stratum 3; 792-81	Organic crust on pot 2153, soluble fraction	−26.0	3910 ± 80	Stäuble (2005)
UtC-2321	House 11, west flanking pit, quadrant r, Stratum 2; 360-33	Organic crust on pot 33, residual fraction	−25.8	7280 ± 100	Stäuble (2005)
UtC-2342	House 11, west flanking pit, quadrant r, Stratum 2; 360-33	Organic crust on pot 33, soluble fraction	−26.0	3060 ± 110	Stäuble (2005)
UtC-2322	House 11, west flanking pit, quadrant v, Stratum 1; 360-80	Organic crust on pot 138, residual fraction	−26.0	7600 ± 80	Stäuble (2005)
UtC-2343	House 11, west flanking pit, quadrant t, Stratum 3; 360-47	Burnt daub fragment, residual fraction	−26.0	4600 ± 190	Stäuble (2005)
**Stadel**					
Erl-18202	Pit	Organic material from inside an earliest LBK sherd	−25.2	6894 ± 66	O’Neill (2013)
**Goddelau**					
Hd-14176	House 1, west flanking pit, surface; 71-5-1	Bovid right proximal metacarpal	−22.2	6370 ± 35	Stäuble (1995, 2005)
OxA-1628	House 3, west flanking pit, quadrant j/f, Stratum 1/2; 9-261/276	Carbonised cereal	−26.0	6300 ± 90	Stäuble (1995, 2005)
Hd-14009	House 3, west flanking pit, quadrant p, Stratum 7; 9-127	*Bos taurus* left part of pelvis	−22.1	6260 ± 40	Stäuble (1995, 2005)
Hd-14173	House 3, west flanking pit, quadrant e, Stratum 7; 9-205	Left distal radius, *Bos primigenius*	−24.3	6295 ± 50	Stäuble (1995, 2005)
KN-3429	House 3, west flanking pit, quadrant j, Schicht 5; 9-308	Unidentified charcoal		6600 ± 85	Stäuble (2005)
KN-3428	House 4, east flanking pit, shovelled surface; 73-4	Unidentified charcoal		0 ± 5	Stäuble (2005)
KN-3430	House 5, west flanking pit, quadrant I, Stratum 3; 12-62	Unidentified charcoal		1730 ± 65	Stäuble (2005)
**Bruchenbrücken**					
Hd-13893	House 2, north pit, quadrant d, Stratum 8; 5-49	Right distal scapula of *Sus scrofa*	−22.4	5970 ± 105	Stäuble (2005)
Hd-13894	House 2, east flanking pit, quadrant bb, Stratum 3; 18-35	Radius/left ulna, domestic pig	−21.4	6005 ± 655	Stäuble (2005)
Hd-14273	House 2, east flanking pit, quadrant bb, Stratum 4; 18-36	Ulna and tibia, domestic pig	−21.2	6235 ± 55	Stäuble (2005)
KI-2598	House 2, north pit, quadrant d, Stratum 5; 5-37	*Quercus* sp. charcoal		6370 ± 90	Stäuble (2005)
KI-2599	House 2, north pit, quadrant g, Stratum 10; 5-98	*Quercus* sp. charcoal		6370 ± 90	Stäuble (2005)
KI-2600	House 2, west flanking pit, quadrant aa, ‘Schicht’; 18-63c	Unidentified charcoal, probably *Quercus* sp.		6390 ± 100	Stäuble (2005)
OxA-1629	House 2, north pit, quadrant h, Stratum 4; 5-92	Carbonised cereal	−26.0	6240 ± 90	Stäuble (2005), Whittle (1990)
OxA-1630	House 2, east flanking pit, quadrant bb, Stratum 4; 18-36	Carbonised cereal	−26.0	6390 ± 100	Stäuble (2005), Whittle (1990)
OxA-1632	House 3, west flanking pit, quadrant m/o/q, Stratum 5/1/all; 150-14/29/61	Carbonised cereal	−26.0	5410 ± 90	Stäuble (2005), Whittle (1990)
OxA-1631	House 6, west flanking pit, quadrant m/o/aa, all layers; 76-134/135/136	Carbonised cereal	−26.0	4700 ± 110	Stäuble (2005), Whittle (1990)
Hd-13895	House 8, pit, quadrant g, Stratum 3; 250-17	Human bone		4030 ± 45	Stäuble (2005)
OxA-1633	House 8, east flanking pit, quadrant c, all layers; 248-29a	Carbonised cereal	−26.0	6190 ± 80	Stäuble (2005), Whittle (1990)
OxA-1634	House 8/9, flanking pit, quadrant b, Stratum 2; 257-27	Carbonised cereal	−26.0	6040 ± 90	Stäuble (2005), Whittle (1990)
Hd-14548	Grave	Rib and vertebrae fragments, human	−21.3	6365 ± 35	Stäuble (2005)
**Steinfurth Bad Nauheim**					
KIA-24642	Feature 2-10 (loam pit)	Cerealia indeterminate		6135 ± 35	Kreuz (2010), Langenbrink and Kneipp (1990)
**Nidderau-Ostheim**					
KIA-27044	Pit 95-3D	*Triticum dicoccum*		6205 ± 40	Kreuz (2010), Gallay and Hansen (2006)
**Bad Camberg-Würges**					
KIA-20181	Pit 6-h-1	*Triticum dicoccum*		6190 ± 45	Kreuz (2010), Schade and Schade-Lindig (2002)
**Eitzum 1**					
H-1487/985	Pit? ‘Point 5’	Unidentified charcoal		6480 ± 210	Kohl and Quitta (1964, 310), giving pers. comm. from H. Schwabedissen; Breunig (1987, 126), Stäuble (2005)
Bln-51a	Pit? ‘found at 0.45 cm depth in residual block, Point 9 easterly, T 0.0’	Pottery, ‘thick-walled, strongly organic-tempered and weakly fired’		6310 ± 200	Kohl and Quitta (1964), first published as Bln-51, with the value given here for Bln-51a
Bln 51b	Pit? ‘found at 0.45 cm depth in residual block, Point 9 easterly, T 0.0’	Pottery, ‘thick-walled, strongly organic-tempered and weakly fired’		6530 ± 100	Kohl and Quitta (1964)
KIA-17411	Pit 26-125	Cerealia indeterminate		5862 ± 30	Kreuz (2010)
**Eilsleben**					
OxA-1624	Longpit? 23-85.2	Unidentified animal bone		6140 ± 90	Whittle (1990)
OxA-1625	Longpit? 23-85.8	Unidentified animal bone		6030 ± 100	Whittle (1990)
OxA-1626	Longpit? 23-85.9	Unidentified animal bone		6070 ± 100	Whittle (1990)
OxA-1627	Longpit 68-86.9	Unidentified animal bone		6190 ± 90	Whittle (1990)
OxA-1623	Longpit?	Unidentified plant remains		2110 ± 80	Whittle (1990)
Bln-1429	Pit 2-74	Pottery		6560 ± 75	Kaufmann (1983, 193), Stäuble (2005)
Bln-1430	Pit 2-74	Unspecified material		6895 ± 60	Kaufmann (1983, 193), Stäuble (2005)

Only radiocarbon dates that are published as having a direct association with Formative or earliest (*älteste*) ceramics have been included. This means that many sites have more radiocarbon dates than have been used in the modelling, but these are either associated with later periods of LBK activity or do not have explicit published associations with the earliest material. In many cases it has been impossible for us to judge the validity of the published association, as sites are not yet published in detail. Sometimes associations cannot be made at the feature or structure level, but rather an entire site is categorised as only containing Formative or earliest LBK ceramics. It should be noted that the association between cultural material and the radiocarbon sample is critical to avoid circular arguments (by which an early LBK date is asserted on the basis of the calibrated radiocarbon result alone—in the third quarter of the sixth millennium cal BC, say—rather than on the basis of the date from a sample directly associated with diagnostic earliest LBK material).

This legacy dataset is inevitably of variable quality. Although over 60% of measurements have been made by AMS, short-life, single-entity samples that can be confidently associated with the use of the feature from which the samples derived are scarce. Over 40% of samples were of unidentified charcoal or waterlogged wood (or of charcoal from long-lived species such as oak and ash) and so may incorporate an old-wood offset. Other samples consist of disarticulated animal bones or single carbonised cereal grains, where it is not known whether the dated material derived from particular concentrations. Such materials might well be residual (older than their contexts) or intrusive (younger than their contexts).

We have attempted to distil reliable chronology from this mess of data by incorporating each result into the model in a way that is appropriate for the dated material:

- Samples of human bone from graves, and short-lived, charred plant remains (including short-life charcoal) from fired features such as hearths, or large concentrations such as coherent dumps in pits, have been incorporated into the models as short-life material likely to be contemporary with the archaeological activity of interest (n = 20)
- Disarticulated animal bones and short-lived charred plant material from pits or postholes have also been included fully in the models, although we consider the archaeological association in this case to be less reliable (n = 25)
- Samples of unidentified charcoal (or charcoal from long-lived species) that have been dated by AMS and so probably comprised a single fragment have been incorporated into the models using the Charcoal Outlier function of OxCal v.4.2 (Dee and Bronk Ramsey 2014). This uses an exponential distribution to reflect the underlying age distribution of a living forest and, assuming that the dated fragments are selected randomly from this forest, incorporates these dates into the models following this distribution. This attempts to allow for the possibility of inbuilt age in the model calculations (n = 23)
- Samples of unidentified waterlogged wood or charcoal (or charcoal from long-lived species) that have been dated by conventional radiometric methods have been incorporated into the models as *termini post quos*, as have two samples where the dated material is not recorded (n = 38)
- A number of samples of bulk pottery which were dated by Gas Proportional Counting by the Berlin laboratory in the early days of the method are modelled as *termini post quos* since the origin of the dated carbon is uncertain (n = 4)
- Two sets of data have been excluded from the models because we consider that there is a high probability that the measurements are inaccurate for technical reasons. Replicate AMS measurements on different chemical fractions of organic crusts on pottery and daub made at the Utrecht facility (on samples from Schwanfeld and Enkingen) show poor reproducibility, and a single result on residue from temper in a sherd from Stadel seems anomalously old. We have thus excluded all results on carbonised residues from the modelling (n = 13). Series of measurements made on bone samples using Gas Proportional Counting at Heidelberg University in the 1980s have (at Schwanfeld and Bruchenbrücken) produced results that are much more widely spread than those from other short-life samples from these sites, which suggests that these results may have been subject to the technical difficulties with dating some low-collagen bone experienced by the laboratory at that time (e.g. at Trebur: Spatz 2001). Since we have no independent evidence of which measurements are accurate, all measurements on bone made at Heidelberg at this time are excluded from the modelling (n = 14)
- Six results on carbonised plant remains (two on single cereal grains from Bruchenbrücken [OxA-1631–2], one on a sample of unidentified plant material from Eilsleben [OxA-1623], one on an indeterminate cereal grain from Eitzum 1 [KIA-17411], and two on bulk samples of unidentified charcoal from Goddelau [KN-3428 and KN-3430]) have been excluded from the models since they are clearly intrusive (or contained a component of recent material)
- It should be stressed that we do not think that the models presented below provide robust estimates for the date of the end of the earliest or *älteste* LBK pottery style; for that, a much better data set is required. For example, the dates now published for Rottenburg-Fröbelweg include several which appear far too late for the earliest LBK or indeed the LBK at all [see Denaire et al. (submitted, fig. 23), by comparison, for a model for the latest LBK in the Rhine valley]. Only earliest LBK pottery has been identified at this site (Bofinger 2005) and there is no apparent reason here to question the measurements on technical grounds, so presumably there is a so-far unresolved problem with the detection of later activity. For this reason, we have excluded all the results from the site from the models, as we have no evidence to determine which associations may be robust (n = 15).

In total, therefore, our models include 110 radiocarbon measurements (with a further 48 not included in the analysis either because there is a significant possibility that the measurements are inaccurate or because we think the dated material was intrusive). Forty-five measurements on short-lived samples are included fully in the models as potentially accurately dating the ceramics with which the dated material was associated (41%); 35 measurements, which were probably made on single fragments of charcoal of uncertain maturity, are included in the models using the charcoal outlier function of OxCal (21%); and 42 measurements, on bulk samples of uncertain maturity or composition, are included in the models as *termini post quos* (38%) (we are aware of four further results from Brunn/Wolfholz site 2a [KI-13612, KI-13615, VERA-1799–1800], and four from Brunn/Wolfholz site 2b [VERA-199, −200, −202 and −1797], apparently associated with Formative LBK pottery, but these are currently inadequately published and cannot be included in the analysis).

The first chronological model for these data is shown in Figs. 18 and 19. This model places all the radiocarbon dates, modelled in accordance with their material and contexts using the methods described above, in a single continuous phase of activity (effectively this represents the currency of longhouses with flanking pits). According to this model, these first appeared in *5525*–*5475* *cal BC* (*25% probability*; *start LBK*; Fig. 18) or *5455*–*5375* *cal BC* (*70% probability*), probably in *5505*–*5485* *cal BC* (*13% probability*) or *5425*–*5385* *cal BC* (*55% probability*). This is clearly earlier than the longhouses at Szederkény.

The second chronological model for these data is shown in Figs. 20 and 21. This model separates the Formative phase of the LBK (following Bánffy and Oross 2009, 2010) and places it earlier than the earliest or *älteste* LBK. This effectively dates both the initial appearance of longhouses with flanking pits and the time of their massive dispersal or diaspora across central Europe. This model suggests that the Formative LBK began in *5625*–*5480* *cal BC* (*95% probability*; *start formative*; Fig. 20), probably in *5565*–*5490* *cal BC* (*68% probability*). The transition from the Formative to the earliest LBK, and thus the expansion of longhouse building, occurred in *5395*–*5320* *cal BC* (*95% probability*; *end formative/start earliest*; Fig. 20), probably in *5375*–*5330* *cal BC* (*68% probability*). It is *89% probable* that the longhouses at Szederkény post-date the beginning of the longhouse diaspora, although only by a period of −*20* to *75* *years* (*95% probability*; distribution not shown), probably *1*–*50* *years* (*68% probability*) (the negative value [−20 years] represents the probability, in this case *11%*, that the longhouses at Szederkény were in fact the first of the diaspora).

The third chronological model for these data is shown in Figs. 22 and 23. This model again separates the Formative phase of the LBK and places it before the earliest LBK. The earliest phase is, however, separated into three regions, in order to investigate the pace of the diaspora. Our divisions are pragmatic. Szederkény has been placed with earliest LBK sites south and east of the bend of the Danube. An eastern group has been defined north of the Danube, and east of Linz, and a western group from further upstream of the Danube and in the Rhine valley (Fig. 1). The division at Linz is entirely pragmatic, splitting a continuum simply on the basis of an apparent spatial division within the sample of earliest LBK sites that currently have radiocarbon dates.

This model suggests that:

- The Formative LBK began in *5610*–*5475* *cal BC* (*95% probability*; *start formative*; Fig. 22), probably in *5545*–*5485* *cal BC* (*68% probability*)
- The Formative period ended in *5445*–*5340* *cal BC* (*95% probability*; *end formative*; Fig. 22), probably in *5420*–*5360* *cal BC* (*68% probability*)
- The earliest LBK began in the southeastern group in *5395*–*5240* *cal BC* (*95% probability*; *start southeast*; Fig. 22), probably in *5365*–*5300* *cal BC* (*68% probability*)
- The earliest LBK began in the defined eastern group in *5410*–*5275* *cal BC* (*95% probability*; *start east*; Fig. 22), probably in *5370*–*5315* *cal BC* (*68% probability*)
- The earliest LBK began in the defined western group in *5415*–*5285* *cal BC* (*95% probability*; *start west*; Fig. 23), probably in *5380*–*5325* *cal BC* (*68% probability*).

**Fig. 24:**
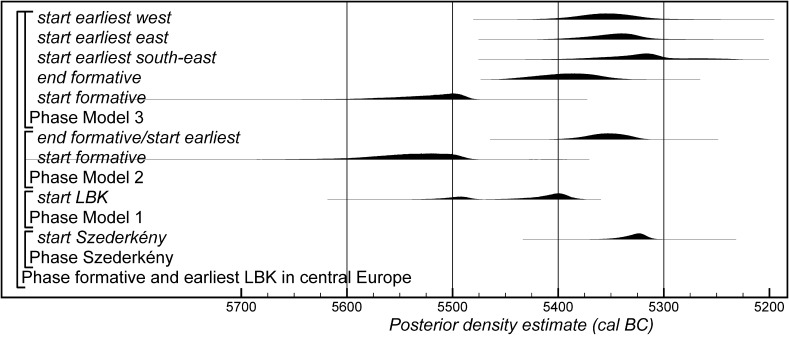
Comparison of key parameters for the Formative LBK and the start of the earliest (*älteste*) LBK from the models defined in Figs. 18–19 (Model 1), Figs. 20–21 (Model 2), and Figs. 22–23 (Model 3), along with the establishment of the settlement at Szederkény (Model 1; Fig. 11)

Figure 24 shows a summary of key parameters from all three models for the first appearance and spread of longhouses. It is clear that the first examples are associated with the Formative phase of the LBK and probably appeared in the decades around 5500 cal BC. The expansion of the distribution of longhouses with flanking pits appears to have begun in the middle part of 54th century cal BC, when in the space of a generation or two they spread across an area of central Europe more than 1000 km across. Given the variable quality of the data currently available, further unpicking the pace and direction of LBK expansion within the 54th century cal BC is hazardous (although it would probably be possible with a concerted attempt to re-date existing archives). The longhouses at Szederkény come out of this process of expansion, but may not belong to its very first decades. People there, however, picked the longhouse with flanking pits out of the LBK repertoire but preferred different pottery technologies and styles. That is unusual, from the point of view of both the Vinča and the LBK worlds, and we go on to think about how our formal modelling affects the kind of narrative which we can now write about these transformations and amalgamations.

## Discussion

### The Settlement at Szederkény in its Regional Setting

The models set out above have suggested the more or less contemporaneous development of a large settlement. That they also indicate a shorter duration for the central part can be supported by the facts that this portion of the site is less densely settled; that there are no superpositions or overlaps between the house-rows, unlike in the eastern and western parts of the settlement; and that there are only a few graves. It can be noted, however, that the position and orientation of burials seem more regular in both the eastern and the central parts (with almost exclusively left-crouched bodies, with an east–west/southeast–northwest orientation), while the western graves show more variation (with some right-crouched bodies, and some north–south orientation).

Rather like the early Neolithic Starčevo occupation of the region, the layout and organisation of LBK settlements in Transdanubia have been characterised by scattered hamlet-like sites both in the Formative and the succeeding phase of the culture (Bánffy and Oross 2009, p. 224; Oross and Bánffy 2009, pp. 177, 180). However, these assumptions were based on a very limited number of excavated sites. The change resulting in large, densely built settlements, as well as in an overall shift in population density and subsistence strategies, did not appear to take place earlier than the start of the later LBK (Bánffy and Oross 2009, p. 224; Oross and Bánffy 2009, pp. 182–184). Balatonszárszó-Kis-erdei-dűlő is a good example where a small site with a limited number of house units grew into a larger settlement (Marton and Oross 2012, p. 225; Oross 2013b, pp. 320–345).

Recent discoveries in southeast Transdanubia, including the sites of Tolna-Mözs (Marton and Oross 2012; Rassmann et al. 2015, pp. 1–4, figs. 2–5); Alsónyék-Bátaszék (Oross et al. in press b); and Versend-Gilencsa (excavated in 2006–2007) provide new insights into the organisation and settlement dynamics of developed LBK sites. Considerable numbers of house plans arranged into rows and separate house clusters have now been found, associated with finds of the early LBK and early Vinča periods in Transdanubia. In contrast to Szentgyörgyvölgy-Pityerdomb (Bánffy 2004), the overall plan of the Formative LBK site of Brunn/Wolfholz 2 (Lenneis 2004, fig. 1; Stadler 2005, fig. 11; Oross 2013b, p. 84, fig. 4.7) suggests that in areas with a higher population density, the intensive occupation of some sites may have started earlier. The seemingly rapid development seen at Szederkény fits this trend nicely. It will be for future research to elaborate and refine models for site formation processes in the whole region between Lake Balaton and the Dráva river.

### Mixture and Amalgamation: Approaches and Terms

Addressing themes of ancestry, generation, substance, memory and land, Tim Ingold (2000) has discussed indigenous attitudes in terms of two competing models: genealogical and relational. In the genealogical model, people are seen as having fixed identities, and ‘culture as a corpus of traditional wisdom, handed down as a legacy from the past’ (Ingold 2000, p. 137). In the relational model, cast in the metaphor of a rhizome rather than that of a tree, identities are performed in engagement with the world, seen as ‘an immense tangle of interlaced trails’ (Ingold 2000, p. 149); it is relationships rather than relatedness that should matter (Ingold 2000, p. 144). Perhaps we should be wary of such large-scale generalisation and such absolute distinctions, since it is possible that different dimensions and facets of identity could be expressed in varying contexts (Bloch 1998). Nonetheless, a relational approach as defined above seems far more promising in the setting of change and mixture described in this paper. It accords too with a general view of social life as something that is continually negotiated and performed, rather than simply enacted (Carrithers 2010; Garfinkel 1988), and that is worked at within sets of relationships which are better characterised as interaction spheres, networks or meshworks (Caldwell 1955; Latour 1993; Ingold 2011) than as static, necessarily bounded entities. This seems all the more attractive in situations of rapid, extensive change and encounter, such as described in this paper for the Carpathian basin, and for central Europe more widely, in the second half of the sixth millennium cal BC.

How best then to catch the tone of what may be going on? A parallel debate on colonial encounter is illuminating. Three strands are particularly relevant. First, an array of ways to characterise mixture has been set out. Matthew Liebmann, for example (2013, 2015), has compared and contrasted the notion of hybridity—which he advocates—with those of acculturation, syncretism, bricolage, creolisation and *mestizaje*. All, in their different ways and with their different connotations and histories of use, are to do with cultural convergence and ‘creation through recombination’ (Liebmann 2013, p. 27), and at a certain level could be seen as synonyms. But acculturation tends to be associated with a checklist approach to separate traits, and syncretism with religious ideas (Clack 2011). Bricolage goes back to Lévi-Strauss (1966) and ‘entails the creative recombination of cultural elements by individuals acting within a limited range of options’ (Liebmann 2013, p. 29). Creolisation begins with recombinations of ‘shared lexical elements in a new grammar and syntax’, and is particularly associated with studies of dislocation and diaspora (Liebmann 2013, pp. 28–29; cf. Eriksen 2007; Knörr 2010). *Mestizaje* addresses the mixing of peoples in colonial encounter, but has been criticised for failing sufficiently to acknowledge indigenous resistance and identity (Liebmann 2013, p. 29; cf. Sauer 2015). Hybridity is advocated partly for its lack of such baggage, partly for its stress on reworking rather than simple recombination of ‘distinct cultural forms’ and partly for an emphasis on issues of power, inequity and resistance (Liebmann 2013, pp. 30–31, 2015, pp. 323–324). In a study of Mississippianisation in the American Bottom, hybridity has been argued to be a process that generates innovation, resulting in ‘the creation of something that may not reference its origins in any obvious way and therefore cannot be reconstituted into those original parts’ (Alt 2006, p. 292). It is seen to occur in ‘a liminal space, a region of overlap where differences can meet and create a new space’, such as in the encounter between people with different traditions’ (Alt 2006, p. 292).

As already noted, however, hybridity raises difficult problems of defining prior purity (Stockhammer 2012), and thus of what is not a hybrid (Palmié 2013; Silliman 2015, 7; cf. Bhabha 1990; Burke 2009). It also presents the question of when hybridity ends (Silliman 2015, p. 7), and tends to be applied more to the colonised than to the coloniser (Silliman 2015, pp. 12–13). Other metaphors and potential replacements for the notion of hybridity, such as entanglement (Hodder 2012), have also been seen as under-theorised (Silliman 2015, p. 15).

Another concept under discussion is that of ethnogenesis (Voss 2015). This emphasises process: ‘ethnicity is something people do, rather than something people are’ (Voss 2015, p. 657). This in turn raises the question of what ethnicity is, which Barbara Voss (2015, p. 658) suggests is a ‘consciousness of difference’, concerned with ‘ideologies of shared and divergent history, ancestry and tradition’. Ethnogenesis again overlaps with the list of other concepts discussed above, but is seen as best applying to ‘situations in which prior modes of identification are transformed and replaced by new identity practices’ (Voss 2015, p. 659); such transformations in social identity are also seen to be spurred by ‘substantive demographic shifts—aggregation, disaggregation, displacement, and migration—combined with the emergence or imposition of new structures of power’ (Voss 2015, p. 666).

This brief review shows how many of the terms available for discussion of cultural combinations carry particular baggage from past usage and can be problematic, and it is tempting to fall back on more general terms such as mixture and amalgamation. What seems at least as important is to emphasise relationships, performance and intersecting networks. With those starting points, what range of narratives can be constructed for what was going on at Szederkény in particular and in the Carpathian basin and central Europe in general, and which might be the most plausible?

### From Formal Modelling to a Choice of Narratives

First, we need to go back to pottery styles, graves and the architecture of the longhouse.

Following Model 1, as set out above (Fig. 11), and contrary to initial typological assessment, it now appears that the various pottery styles across what we take to be the single, large settlement of Szederkény were contemporary, though it does seem that the western part of the site was abandoned last. That prompts further reflection on what was shared and what was distinct.

There is an important shared tradition across the styles in question. This shared tradition and style involve the technique of pottery making, such as firing and surface treatment techniques. Among these features, the most apparent are the black burnishing and the shiny red slip, applied to the pedestals and the lower parts of vessels, and usually combined with an unoxidised area on the upper part of vessels, which results in the so-called ‘black-topped–red-slipped’ pottery. The black-topped pottery, being a very specific feature that needed highly specialised know-how in pottery-making and pyrotechnology (Kaiser 1984, p. 253), is present both in Vinča A and the Ražište style.

But there are also important differences, and so even within the ceramic repertoire of neighbouring parts of the settlement we are confronted with contemporaneous material diversity. Forms only present in the Vinča A style include sharply biconical bowls (Fig. 8: 1–3, 5; Fig. 10: 3–6), often in pedestalled versions (Fig. 8: 4, Fig. 10: 1) and with a thickened shoulder around the carination (Fig. 8: 4, 6–7; Fig. 10: 1–2). Smoothing and light channelling on the shoulder are often found on these vessels (Fig. 8: 2; Fig. 10: 3–5). These forms and surface treatment techniques can be considered the most distinctive features of the Vinča A style. Both styles have incised decorative motifs filled with stabbed incisions (in Vinča A style, see Fig. 8: 11, 13; Fig. 10: 6). Such stabbed decoration with curvilinear motifs and on the upper part of vessels only appears, however, on Ražište vessel surfaces (Fig. 9: 4–5, 8–11). A further, related difference is that houses with Vinča A pottery, mainly in the eastern settlement segment, used a great number of small clay figurines and miniature altarpieces, but not one of these can be found in houses with the Raziste-style pottery (Jakucs and Voicsek 2015, fig. 20–1).

The sporadic occurrence of LBK-style sherds is a complicating puzzle. In Ražište contexts, there are some sherds which are mostly typical of more developed LBK phases, perhaps reflecting the presumed longer duration of the Ražište style. In the eastern part of the settlement with households characterised by early Vinča pottery, these sherds with LBK characteristics are always part of the coarse-ware assemblage. Grave 237 is especially interesting, as it is strongly suggestive of composite identity within one household. Here the skeleton was accompanied by a globular vessel with a cylindrical neck and decorated with an incised spiral motif, which can be compared to early LBK style elsewhere. The incised spiral meander motif on the storage vessel from the burial can best be likened to the ceramic styles of the early central European LBK (Bicske-Bíňa and Milanovce), although it remained a popular motif until the Notenkopf period (Marton 2008; Pavúk and Farkaš 2013). Fragments of vessels with similar decoration, although quite rare, were also found in the eastern and central parts of the settlement. The burial was found in the western longpit of house H12, one of the earliest of the Szederkény features, with exclusively Vinča A pottery (Jakucs and Voicsek 2015, fig. 11).

Such ceramic diversity is accompanied by the presence of both graves and longhouses. At present, as noted above, it is unprecedented to find settlement burials in the early Vinča orbit, though they are in themselves unremarkable as a feature of developed LBK sites, including in Transdanubia. Equally—if not more—striking is the fact that the inhabitants of Szederkény, on all parts of the site, constructed houses with all the elements regarded as a hallmark of an LBK longhouse. The three formal models presented above (Figs. 18–19, 20–21, and 22–23; summarised in Fig. 24) now allow a more robust estimation of the appearance and development of longhouses in general, and the chronological position of the Szederkény longhouses in particular in relation to that process. These two facets of the modelling both demand comment.

Clearly what our models suggest has many implications for the whole shape and character of LBK development, and requires much further discussion elsewhere. At this stage, it is worth stressing two key points. First, while the formal estimates given for the start of the Formative phase in Transdanubia and eastern Austria broadly conform with the majority of informal estimates for the start of the LBK as a whole, that is, in the decades around 5500 cal BC, those for the start of the earliest LBK are significantly later than conventional wisdom suggests, placing the LBK diaspora not earlier than the 54th century. Among the many implications which will have to be discussed elsewhere is the effect this has on our view of the rate of growth of the developed LBK. Secondly, recent and ongoing aDNA studies have strongly revived the older concept that the spread of the longhouses into central Europe went along with the spread of new people (among others: Brandt et al. 2013, 2014). The data also suggest gene-pool shifts as well as continuities within the Carpathian basin in the middle of the sixth millennium cal BC, between Starčevo and LBK (Szécsényi-Nagy et al. 2014, 2015). But the geographical spread of such analyses is incomplete, and there is no reason yet wholly to abandon arguments that the indigenous population was also involved in processes of transformation (Brandt et al. 2014, p. 101). In any case, unless indigenous populations had somehow died out before the LBK diaspora, not only transformation but considerable disruption is strongly implied, and it is to such a scenario of change in the 54th century cal BC, now formally modelled (as opposed to being merely asserted) as rapid, that the amalgamations visible at Szederkény belong. Though there is no particular need to think in terms of specific ethnicities, the situation does recall the discussion of ethnogenesis noted above, and its frequent attendant conditions of demographic shift (Voss 2015, p. 666).

This also opens up a choice of narratives for the developments and combinations seen at Szederkény. At a general level, in the area of already established Neolithic settlement, larger and more visible settlements began to appear, with more people living together than had been the case at the vast majority of earlier sites. In the regions beyond the previous limits of Neolithic settlement, larger and more numerous settlements also appeared, typified here by the longhouse diaspora. Material culture changed too, the sets of things and practices which we label as Vinča and LBK replacing those we label as Starčevo. It is easy, following the kind of chronology conventionally constructed by a combination of culture history and informal inspection of radiocarbon dates and familiarly presented in chest-of-drawers fashion, with block piled neatly upon successive block, to think in terms of simple processes of replacement, and to suppose that total distributions at the end or peak of later development should speak for all stages of long processes of change. But why should this have been so? There need have been nothing predetermined, in the 54th century cal BC, about the later distribution, boundaries and development of the Vinča and LBK orbits, and the biography of the Szederkény settlement speaks to the fluidity and porosity of identities in the conditions of change starting in the 55th and 54th centuries cal BC.

More specific hypotheses can also be entertained. First, we could posit that people of basically local descent, caught up in processes of rapid change in the 54th century cal BC, sought to consolidate new household and community identities by adopting new material practices—longhouses from Transdanubia and beyond to their north, and pottery of their own or regional invention. That later on longhouses were distinctively associated with the developed LBK world and black-topped pottery with the Vinča orbit is irrelevant to the local and regional conditions of change in the 54th and 53rd centuries cal BC which are now becoming visible. Secondly, we could envisage some movement and amalgamation of people in the conditions of change and demographic shift in the 54th century cal BC. With Transdanubia and regions to its north and northwest rapidly beginning to be settled, some people could have come south to found a new settlement in an area with previously scarce Starčevo settlement, following the new social vogue for longhouses, while others could have come north from the emergent Vinča world. At this time, what were later to become separate cultural spheres were interleaved, and this is another reason perhaps for being suspicious of terms like *hybridity* for labelling the emergent combinations and mixtures of people, things and practices. It is we who risk being fettered by notions of static, fixed identities. Given the present state of the evidence, it is hard to choose between these two hypotheses; they could indeed be combined. Two glosses on these scenarios could also be considered: the theoretical possibility that the development of the first longhouses goes back to late Starčevo groups in northern Croatia and southern Transdanubia; and that such an emergence occurred over a wider area, between say Lake Balaton and the Drava river, opening the way for different kinds of recombination between a local population familiar at least with some elements of building traditions and receptive to other new things and practices from both north and south. The frustrating incompleteness of the remains from Alsónyék was noted earlier, and these speculations require much more evidence, not least about late Starčevo architecture, from the research which continues in this area.

## Conclusions

The more precise timing afforded by formal modelling of the radiocarbon dates from Szederkény in southeast Transdanubia casts new light on a series of interlinked questions. It reveals the biography of this substantial settlement in more detail than preliminary typological analysis, and establishes the contemporaneity of different ceramic styles: Vinča, Ražiste and LBK. The Vinča A pottery at Szederkény is at least as early as anything else known in the Vinča complex. The site probably began in the later 54th century cal BC and lasted until the early 52nd century cal BC; occupation probably started first in the eastern and western parts of the site, and was probably abandoned last in the western part.

Formal modelling also enables the appearance of longhouses at Szederkény to be set within the wider history of longhouse emergence in the Formative LBK of Transdanubia and eastern Austria, from c. 5500 cal BC, and rapid longhouse diaspora, in the earliest LBK of central Europe, probably beginning in the middle of the 54th century cal BC.

The formal chronological approach presented here further serves to demystify previously rather vague discussion of origins and shifts in material culture patterning, and to direct debate instead to the fluid circumstances of rapid change in which new practices, performances, combinations and amalgamations emerged. Specific narratives for either purely local development or change brought in part by outsiders can be suggested, though further evidence is required before a definitive story can emerge, and that serves to define future goals for ongoing research in this highly significant area.
